# Sphingosine-1-phosphate ameliorates necrotizing enterocolitis by regulating mitochondrial redox homeostasis and gut microbiota

**DOI:** 10.1038/s41419-026-09144-8

**Published:** 2026-07-31

**Authors:** Wenqiang Sun, Yihui Li, Xue Liu, Minqian Zhou, Huawei Wang, Haifeng Geng, Wenmei Li, Zhirong Xie, Lili Li, Xinyun Jin, Xueping Zhu

**Affiliations:** 1 Department of Neonatology, Children’s Hospital of Soochow University, Suzhou, China; 2 Suzhou Medical College, Soochow University, Suzhou, China; 3 Department of Neonatology, The Affiliated Suzhou Hospital of Nanjing Medical University, Suzhou, China

**Keywords:** Experimental models of disease, Biomarkers

## Abstract

Sphingolipid metabolism critically links intestinal barrier integrity to inflammatory responses, and its role in necrotizing enterocolitis (NEC) pathogenesis may inform strategies for prevention and treatment. To investigate this association, metabolomic profiling of serum samples from preterm infants was performed, and the findings were validated in independent cohorts. The effects of sphingosine-1-phosphate (S1P) were evaluated in LPS-induced IEC-6 cells and experimental NEC models. Mechanistic studies were performed using pharmacological inhibitors, and microbiota-dependent effects were assessed through bacterial intervention experiments. Serum S1P levels were reduced prior to NEC diagnosis and correlated with disease severity. Opaganib reduced S1P levels and exacerbated NEC pathology, while S1P supplementation reversed these effects. Mechanistic investigations revealed that S1P exerted a protective effect against NEC by modulating mitochondrial oxidative respiration and redox balance, as well as by reshaping the gut microbiota to attenuate inflammation, apoptosis, and intestinal barrier disruption. The protective effects of S1P were partially attenuated by S1PR1 blockade. In addition, supplementation with *Bacteroides thetaiotaomicron* improved sphingolipid metabolic profiles in NEC intestinal tissues and upregulated S1P levels. These findings suggest that dysregulation of sphingolipid metabolism is associated with NEC and intestinal barrier dysfunction. S1P may alleviate NEC-related injury in experimental models, potentially involving S1PR1 signaling, mitochondrial redox balance, and interactions with the gut microbiota.

## Introduction

Necrotizing enterocolitis (NEC) remains one of the most critical gastrointestinal emergencies encountered in neonatal intensive care units, especially for extremely preterm infants, with persistently elevated morbidity and mortality rates [1–3]. Currently, the primary approach for NEC remains medical management; however, surgical intervention is often required for severe cases or when conservative treatment fails [4]. Additionally, survivors frequently suffer from serious long-term sequelae, including short bowel syndrome, growth retardation, and neurodevelopmental impairments [2–5]. Therefore, identifying novel biomarkers for early prediction or therapeutic intervention represents a critical unmet need in current clinical practice.

The clinical presentation of NEC is highly heterogeneous, and its pathogenesis involves complex, multifactorial, and multisystem interactions, including intestinal barrier disruption, dysregulated immune activation, microbial dysbiosis, and metabolic disturbance [4–7]. Recent studies have shown that significant metabolic disturbances often occur in neonates with NEC even prior to disease onset. These abnormalities impair intestinal barrier homeostasis and contribute to the physiological and pathological progression of NEC [8,9]. Several studies have suggested the potential of supplementing specific metabolites to intervene in the progression of NEC, as preliminarily reported in several animal experiments [8,10]. Although current evidence largely stems from single-center, small-scale studies and requires further validation, these findings imply that targeting host metabolic disturbances at an early stage may offer new avenues for the prevention and treatment of NEC.

Previous clinical omics analyses from our group revealed a significant decrease in serum sphingosine-1-phosphate (S1P) levels in preterm infants prior to the onset of NEC. We aim to employ both cellular and animal models to elucidate the protective mechanism of S1P in NEC through multifaceted investigations encompassing functional validation, microbiota modulation, and transcriptional regulation. The research is expected to provide novel insights into the pathogenesis of NEC and establish a theoretical foundation for metabolic intervention and precise prevention strategies in preterm infants.

## Results

### Reduced plasma S1P is associated with NEC onset in preterm infants

**Fig. 1:**
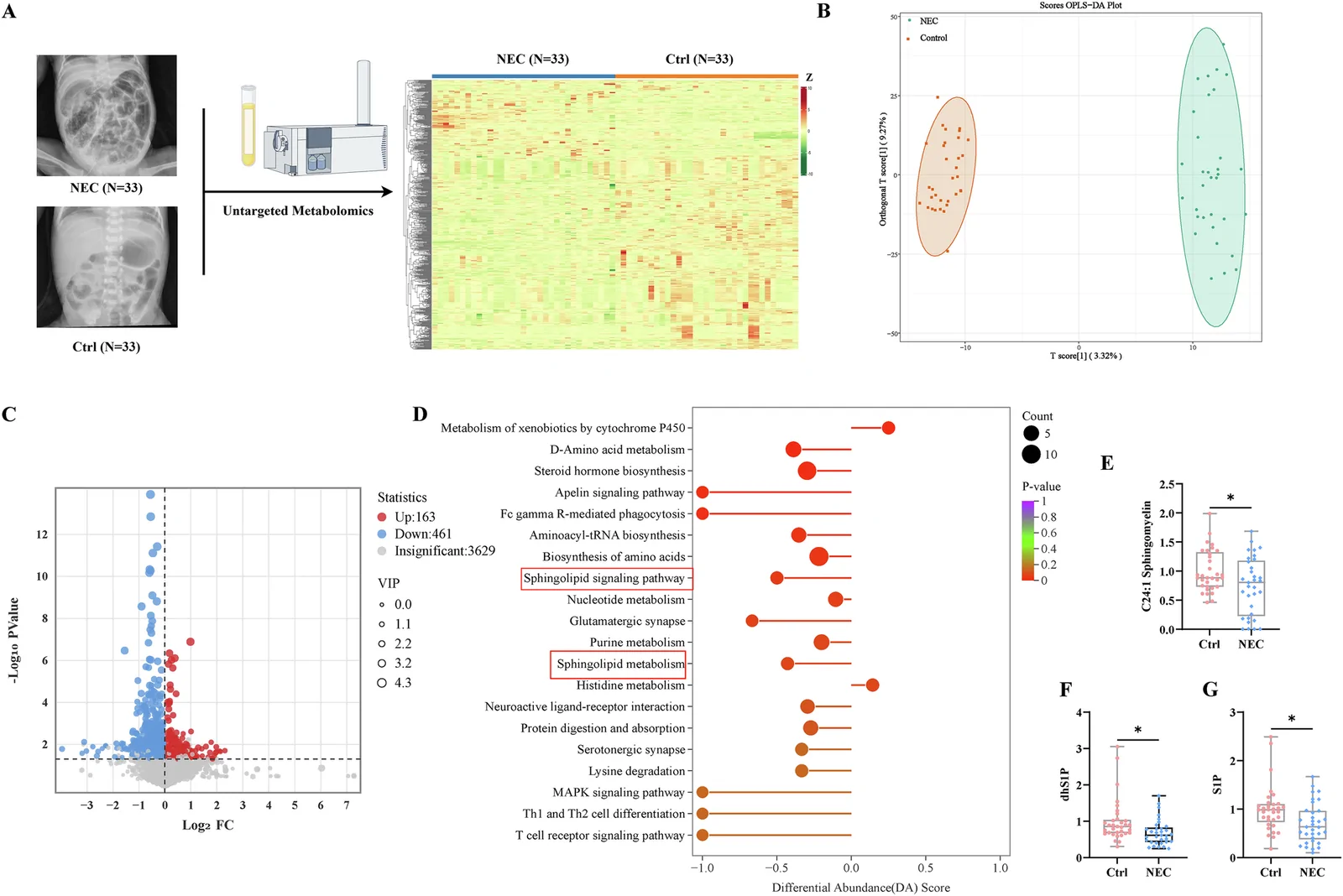
Sphingolipid metabolic dysregulation occurs before NEC onset in preterm infants. **A** Workflow of plasma metabolomic analysis in preterm infants and a heatmap showing the distribution of identified metabolites. Created with Figdraw (figdraw.com). **B** Orthogonal partial least squares-discriminant analysis (OPLS-DA) demonstrating clear metabolic separation between the two groups. **C** Volcano plot analysis identifying a total of 624 differential metabolites between groups, of which 163 were significantly upregulated, and 461 were significantly downregulated in the NEC group. **D** Kyoto Encyclopedia of Genes and Genomes (KEGG) pathway enrichment map of differential metabolites. **E** Expression levels of C24:1 sphingomyelin between the two groups. **F** Expression levels of sphinganine 1-phosphate (dhS1P) between the two groups. **G** Expression levels of sphingosine 1-phosphate (S1P) between the two groups.

Untargeted metabolomics was performed to assess plasma profiles of neonates with NEC (*n* = 33) and healthy controls (*n* = 33), with baseline clinical characteristics summarized in Table S1. Distinct metabolic signatures were observed between the two groups (Fig. 1A–C). KEGG enrichment analysis of differential metabolites revealed significant involvement of sphingolipid metabolism and sphingolipid signaling pathways (Fig. 1D). Notably, NEC patients exhibited marked alterations in sphingolipid-related metabolites, including C24:1 sphingomyelin, sphinganine 1‑phosphate (dhS1P), and S1P (Fig. 1E–G). S1P has been previously implicated in regulating cell proliferation, survival, inflammation, and barrier function [11].

**Fig. 2:**
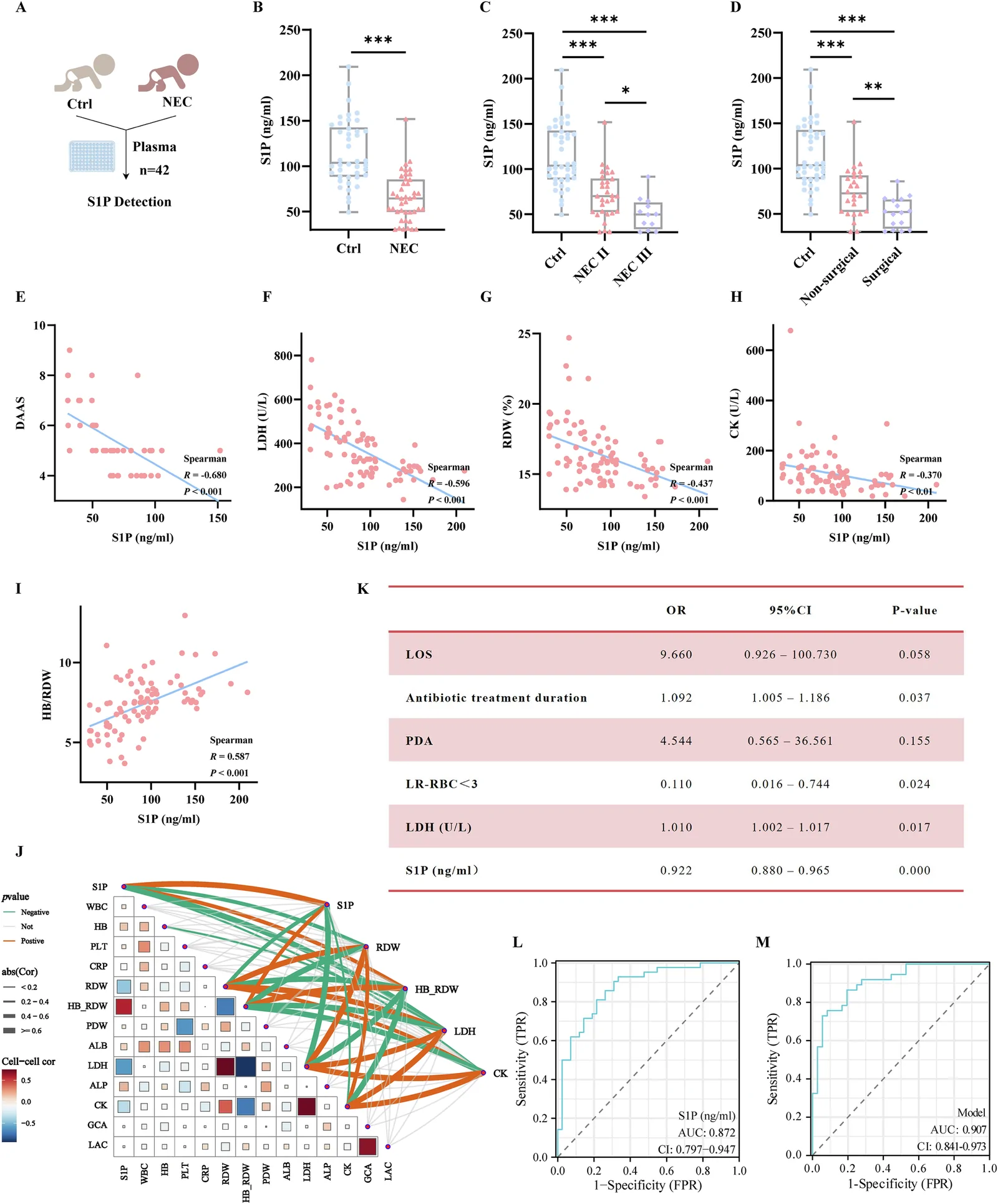
Plasma sphingosine-1-phosphate (S1P) is independently associated with necrotizing enterocolitis (NEC) occurrence. **A** Schematic representation of clinical sample collection and plasma S1P ELISA measurement. Created with Figdraw (figdraw.com). **B**–**D** Group comparisons of plasma S1P levels. **E**–**I** Correlation analyses between plasma S1P and other biomarkers. **J** Summary correlation map of S1P and clinical variables. **K** Analysis of independent risk factors for NEC occurrence. **L** Receiver operating characteristic (ROC) curve predicting NEC occurrence based on plasma S1P levels. **M** ROC curve predicting NEC occurrence based on plasma S1P combined with other risk factors. *P < 0.05, **P < 0.01, ***P < 0.001.

To investigate the clinical relevance of S1P in NEC pathogenesis, we measured plasma S1P levels in preterm infants (gestational age <32 weeks) within 72 h before NEC onset using enzyme-linked immunosorbent assay (ELISA). The NEC group was compared with a matched control group (*n* = 42 each) (Fig. 2A). The mean age at NEC onset was 24 ± 13 days, and maternal and demographic characteristics were comparable between groups. Compared with controls, infants with NEC exhibited significantly higher rates of patent ductus arteriosus, late-onset sepsis, prolonged antibiotic exposure, and elevated lactate dehydrogenase (LDH), as well as markedly lower levels of hemoglobin/red cell distribution width (RDW) ratio, and plasma S1P (all *P* < 0.05) (Table 1, Fig. 2B). Subgroup analysis revealed that plasma S1P levels were further decreased in infants with stage III NEC and in those requiring surgical intervention (Fig. 2C, D). Although partial overlap in S1P levels between groups was observed, a supplementary analysis in a more homogeneous subgroup with minimized confounding factors showed a clearer separation between the NEC (*n* = 15) and control groups (*n* = 15) (Table S2). Spearman correlation analysis demonstrated significant negative correlations between S1P levels and DAAS (*r* = −0.680, *P* < 0.001), LDH (*r* = −0.596, *P* < 0.001), RDW (*r* = −0.437, *P* < 0.001), and creatine kinase (CK) (*r* = −0.370, *P* < 0.01) and a positive correlation with hemoglobin/RDW ratio (*r* = 0.587, *P* < 0.001) (Fig. 2E–J).

**Table 1:** Clinical characteristics, underlying diseases, and main treatments of the study cohort.

Characteristics	NEC (*n* = 42)	Non-NEC (*n* = 42)	*P*
**Maternal characteristics**			
Maternal age, years, median (P25, P75)	30.00 (28.00, 34.00)	30.00 (27.00, 32.00)	0.453
Assisted reproductive technology (*n*, %)	5 (11.90)	6 (14.29)	0.746
Gestational hypertension (*n*, %)	13 (30.95)	8 (19.05)	0.208
Gestational diabetes mellitus (*n*, %)	14 (30.33)	11 (26.19)	0.474
PROM > 18 h (*n*, %)	11 (26.19)	4 (9.52)	0.087
Chorioamnionitis (*n*, %)	1 (2.38)	1 (2.38)	1.000
Antenatal antibiotics (*n*, %)	13 (30.95)	11 (26.19)	0.629
Antenatal MgSO_4_ (*n*, %)	19 (45.24)	17 (40.48)	0.659
Antenatal steroids (*n*, %)	33 (78.57)	33 (78.57)	1.000
Cesarean section (*n*, %)	18 (42.86)	17 (40.48)	0.825
**Infant characteristics**			
Female (*n*, %)	22 (52.38)	24 (57.14)	0.661
Gestational age (GA), weeks	29.66 ± 2.15	29.72 ± 1.97	0.886
Birth weight, g	1173.10 ± 241.94	1129.05 ± 228.25	0.393
Small for GA (*n*, %)	15 (35.71)	12 (28.57)	0.483
**Underlying diseases and comorbidities**			
Respiratory distress syndrome (*n*, %)	25 (59.52)	28 (66.67)	0.498
Patent ductus arteriosus (*n*, %)	22 (52.38)	13 (30.95)	0.046
Atrial septal defect (*n*, %)	24 (57.14)	22 (52.38)	0.611
Ventricular septal defects (*n*, %)	2 (4.76)	1 (2.38)	1.000
Late-onset neonatal sepsis (*n*, %)	17 (40.48)	8 (19.05)	0.032
**Main treatment**			
Antibiotic (*n*, %)	42 (100.00)	39 (92.86)	0.078
Duration of antibiotic use, days	21.00 (11.50, 32.50)	12.50 (8.00, 19.50)	0.004
Mechanical ventilation (*n*, %)	13 (30.95)	8 (19.05)	0.208
Days of mechanical ventilation, days	0 (0, 3)	0 (0, 0)	0.210
RBC transfusions (*n*, %)	32 (76.19)	26 (81.25)	0.157
Number of RBC transfusions <3 (*n*, %)	21 (50.00)	30 (71.43)	0.044
**Laboratory tests**			
White blood cell counts, ×10^9^/L	10.43 ± 5.53	10.77 ± 3.83	0.744
Platelet counts, ×10^9^/L	255.00 (182.25, 362.75)	304.00 (204.50, 417.50)	0.086
Platelet distribution width, %	13.86 ± 2.67	13.82 ± 3.10	0.956
Mean platelet volume, fL	11.25 ± 1.11	11.41 ± 1.11	0.540
Plateletcrit, %	0.33 ± 0.16	0.36 ± 0.13	0.307
HB, g/L	114.83 ± 22.25	121.89 ± 20.55	0.138
Hematocrit, L/L	0.34 ± 0.07	0.36 ± 0.06	0.123
RDW, %	16.28 ± 2.03	15.90 ± 1.41	0.327
HB/RDW ratio	6.76 ± 1.74	7.95 ± 1.45	0.001
C-reactive protein, mg/L	17.35 (1.68, 36.43)	15.32 (3.46, 32.85)	0.673
Albumin, mg/L	31.10 ± 3.13	32.61 ± 3.69	0.083
Total bilirubin	87.70 ± 55.73	96.54 ± 59.81	0.553
Alkaline phosphatase	270.46 ± 146.20	276.83 ± 131.59	0.845
Creatine kinase	116.88 ± 100.44	98.78 ± 69.70	0.357
Lactate dehydrogenase	417.92 ± 140.18	347.27 ± 113.94	0.021
Lactic acid	3.33 ± 2.47	2.68 ± 1.41	0.259
Glucocholic acid	13.41 ± 12.29	10.16 ± 6.48	0.165
Sphingosine 1-phosphate	64.57 (49.59, 85.43)	103.78 (88.45, 142.58)	<0.001

*NEC* necrotizing enterocolitis, *PROM* prelabor rupture of membranes, *RBC* red blood cell, *HB* hemoglobin, RDW red blood cell distribution width.

Multivariate logistic regression analysis identified duration of antibiotic use (OR = 1.092, 95%CI: 1.005–1.186, *P* = 0.037) and elevated plasma LDH levels (OR = 1.010, 95%CI: 1.002–1.017, *P* = 0.017) as independent risk factors for NEC. In contrast, fewer than three red blood cell transfusions (OR = 0.110, 95%CI: 0.016–0.744, *P* = 0.024) and higher plasma S1P levels (OR = 0.922, 95%CI: 0.880–0.965, *P* < 0.001) were protective factors (Fig. 2K). ROC analysis showed that plasma S1P exhibited a moderate discriminative ability between NEC and control groups (AUC = 0.872, 95%CI: 0.797–0.947, *P* < 0.001). When combined with other clinical variables (duration of antibiotic use, RBC transfusions <3, and LDH levels), the predictive performance improved (AUC = 0.907, 95%CI: 0.841–0.973). (Fig. 2L, M).

Taken together, these findings suggest that decreased plasma S1P is associated with NEC, but due to inter-individual variability, it may serve best as a complementary biomarker within a multi-parameter predictive framework, rather than a standalone diagnostic indicator.

### S1P ameliorates NEC in the murine model by restoring mitochondrial morphology and reducing oxidative stress

It is well established that the generation of S1P is primarily catalyzed by two key enzymes: sphingosine kinase 1 (Sphk1) and sphingosine kinase 2 (Sphk2) [12]. In this study, ELISA analysis revealed a significant decrease in Sphk2 protein expression in the intestinal tissues of NEC mouse models compared with controls. Consistently, Sphk2 enzymatic activity assays demonstrated a marked reduction in Sphk2 activity in the NEC group (*P* < 0.05, Fig. S1). Following oral administration of S1P in neonatal mice, intestinal metabolomic analysis revealed time-dependent alterations in the global metabolic profile, with sustained enrichment of sphingolipid metabolism and signaling pathways across multiple time points (Fig. S2). At the metabolite level, S1P and its related molecules exhibited dynamic, time-dependent changes without evidence of sustained accumulation. Notably, S1P showed a transient increase primarily in the intestine, returning toward baseline after 3 h, while plasma levels remained largely stable (Fig. S3); Furthermore, S1P levels were markedly increased in intestinal epithelial cells (IECs) following S1P gavage, accompanied by alterations in S1P-related metabolites, providing evidence that exogenous S1P reaches and accumulates within IECs (Fig. S4). This suggests that the reduction in endogenous S1P production caused by inhibition of Sphk2 activity may be involved in the development of NEC.

**Fig. 3:**
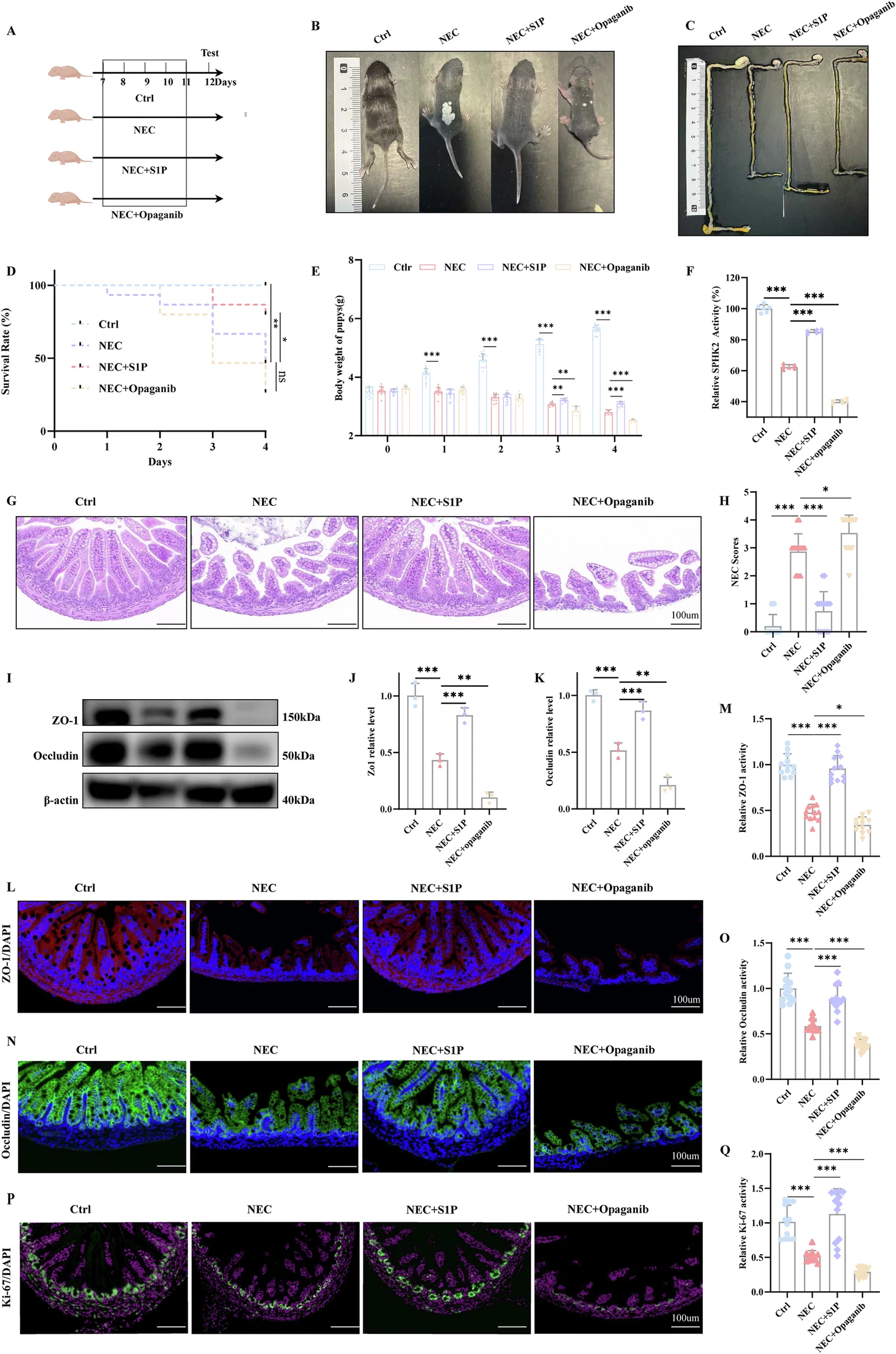
Sphingosine-1-phosphate (S1P) gavage significantly improves necrotizing enterocolitis (NEC) in mice. **A** Schematic representation of NEC modeling and S1P/opaganib gavage intervention. Created with Figdraw (figdraw.com). Representative images (**B**), intestinal morphology (**C**), survival curves (**D**), and body weight changes (**E**) in mice. **F** Determination of Sphk2 enzyme activity in mice intestinal tissue. **G**, **H** Hematoxylin and eosin staining and histopathological scores of distal ilial sections. **I**–**K** Western blot analysis and quantification of the intestinal barrier proteins ZO-1 and Occludin. Immunofluorescence (IF) staining and quantitative analysis of ZO-1 (**L**, **M**), Occludin (**N**, **O**), and Ki67 (**P**, **Q**). *P < 0.05, **P < 0.01,***P < 0.001.

Given the strong clinical association between reduced plasma S1P levels and NEC onset in preterm infants, we next explored whether S1P acts as a protective or pathogenic factor in NEC progression. NEC mice were administered exogenous S1P or the sphk2 inhibitor opaganib via oral gavage (Fig. 3A). Following NEC induction, mice in the NEC group exhibited typical NEC-related symptoms, whereas those treated with S1P showed marked clinical improvement, approaching control group levels. In contrast, opaganib-treated mice displayed exacerbated clinical manifestations (Fig. 3B). Gross anatomical examinations demonstrated that S1P treatment significantly preserved intestinal integrity and length compared to NEC mice, while opaganib worsened intestinal damage (Fig. 3C). Mortality analysis revealed a significant reduction in death rate in the S1P group and a notable increase in the opaganib group compared with NEC alone (Fig. 3D). Regarding weight gain, NEC mice exhibited impaired growth, with significantly reduced body weight at the study endpoint compared with controls. S1P treatment partially rescued this decline, whereas opaganib exacerbated weight loss (Fig. 3E). Sphk2 activity was reduced in NEC and was partially restored by S1P, whereas opaganib further exacerbated this reduction (Fig. 3F). H&E staining confirmed severe intestinal injury in NEC mice, which was substantially alleviated by S1P but worsened following Sphk2 inhibition with opaganib (Fig. 3G, H). Western blot showed that S1P significantly restored the NEC-induced disruption of intestinal barrier proteins ZO-1 and Occludin, whereas opaganib markedly exacerbated this impairment (Fig. 3I–K). IF staining showed that S1P significantly upregulated ZO-1 and occludin, and increased the proportion of Ki67-positive cells in the crypts. These effects were significantly suppressed by opaganib treatment, indicating disruption of epithelial regeneration and barrier integrity (Fig. 3L–Q).

**Fig. 4:**
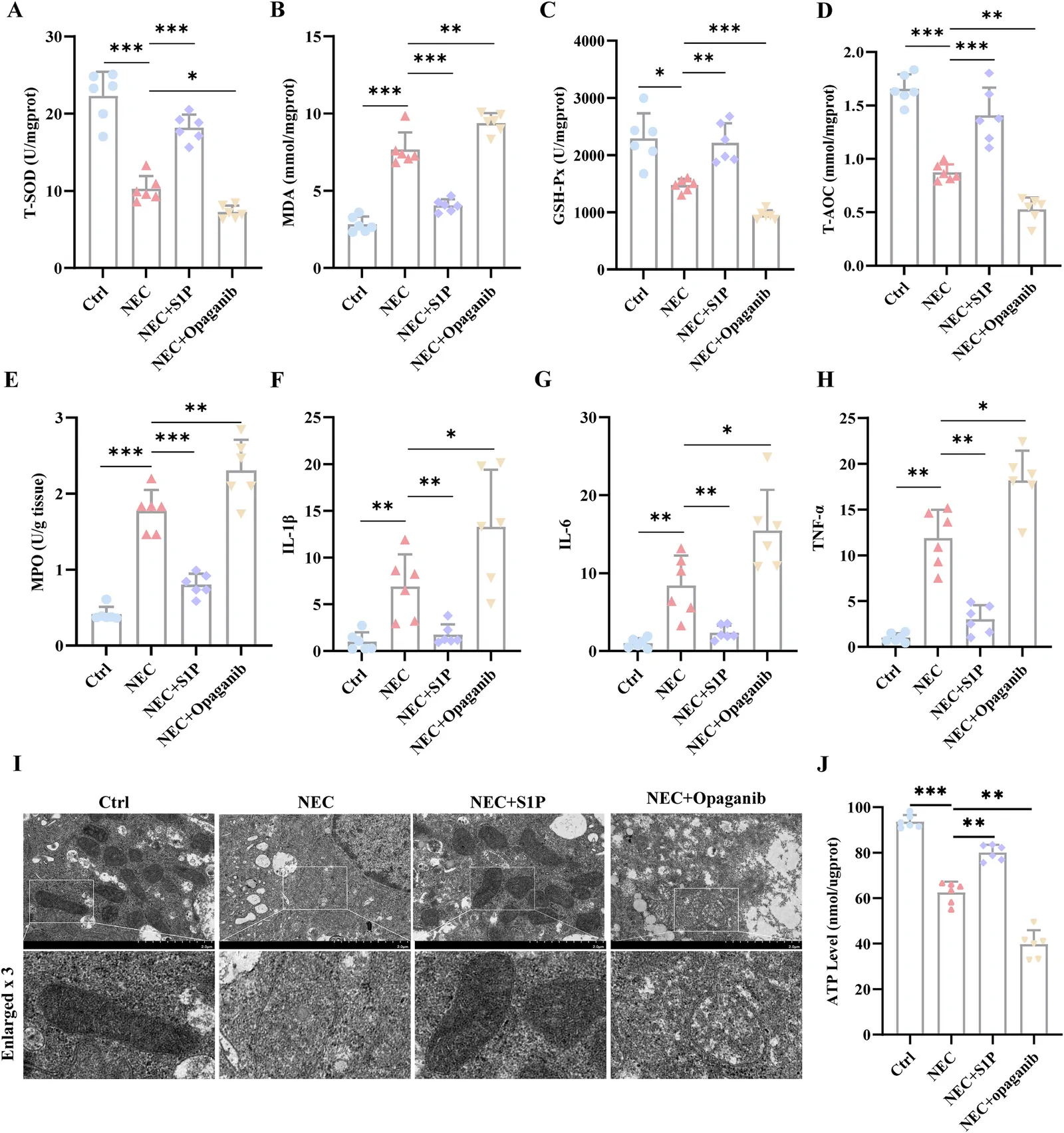
Sphingosine-1-phosphate (S1P) gavage improves intestinal redox homeostasis and mitochondrial morphology while reducing pro-inflammatory markers in mice. Analysis of redox-related markers in mouse intestinal tissues (total superoxide dismutase [T-SOD] (**A**), glutathione peroxidase [GSH-Px] (**B**), total antioxidant capacity [T-AOC] (**C**), malondialdehyde [MDA] (**D**), and myeloperoxidase [MPO] (**E**)). RT-qPCR analysis of pro-inflammatory markers (interleukin [IL]-1β (**F**), IL-6 (**G**), and tumor necrosis factor alpha [TNF-α] (**H**)) in mouse intestinal tissues. **I** Transmission electron microscope observation of mitochondrial morphology in the distal ileum tissue of mice. **J** Measurement of ATP levels in intestinal tissue. *P < 0.05, **P < 0.01,***P < 0.001.

Previous studies demonstrated that S1P regulates mitochondrial function and cellular antioxidant defense mechanisms [13]. Moreover, mitochondrial dysfunction and oxidative stress are central mechanisms in NEC pathogenesis [1,14]. The results of ELISA and RT-qPCR analyses in the present study revealed significantly decreased T-SOD, GSH-Px, and T-AOC and markedly elevated MDA, MPO, and pro-inflammatory cytokines IL-1β, IL-6, and TNF-α in NEC mice. S1P treatment significantly restored antioxidant enzyme levels and reduced oxidative and inflammatory markers, while opaganib exerted opposite effects (Fig. 4A–H). TEM of intestinal tissues showed that NEC-induced marked mitochondrial damage, characterized by irregular morphology, swelling, matrix rarefaction, cristae loss, and structural disorganization. S1P treatment markedly improved mitochondrial ultrastructure, while opaganib treatment aggravated mitochondrial swelling, cristae rupture, and vacuolization (Fig. 4I). ATP measurement in intestinal tissues showed that S1P significantly reversed the NEC-induced decrease in small intestinal ATP levels, whereas opaganib treatment markedly exacerbated this reduction (Fig. 4J). These results collectively demonstrated that the Sphk2–S1P signaling axis plays a protective role in NEC by maintaining mitochondrial homeostasis and mitigating oxidative stress.

### S1P partially modulates NEC-associated intestinal microbiota composition

**Fig. 5:**
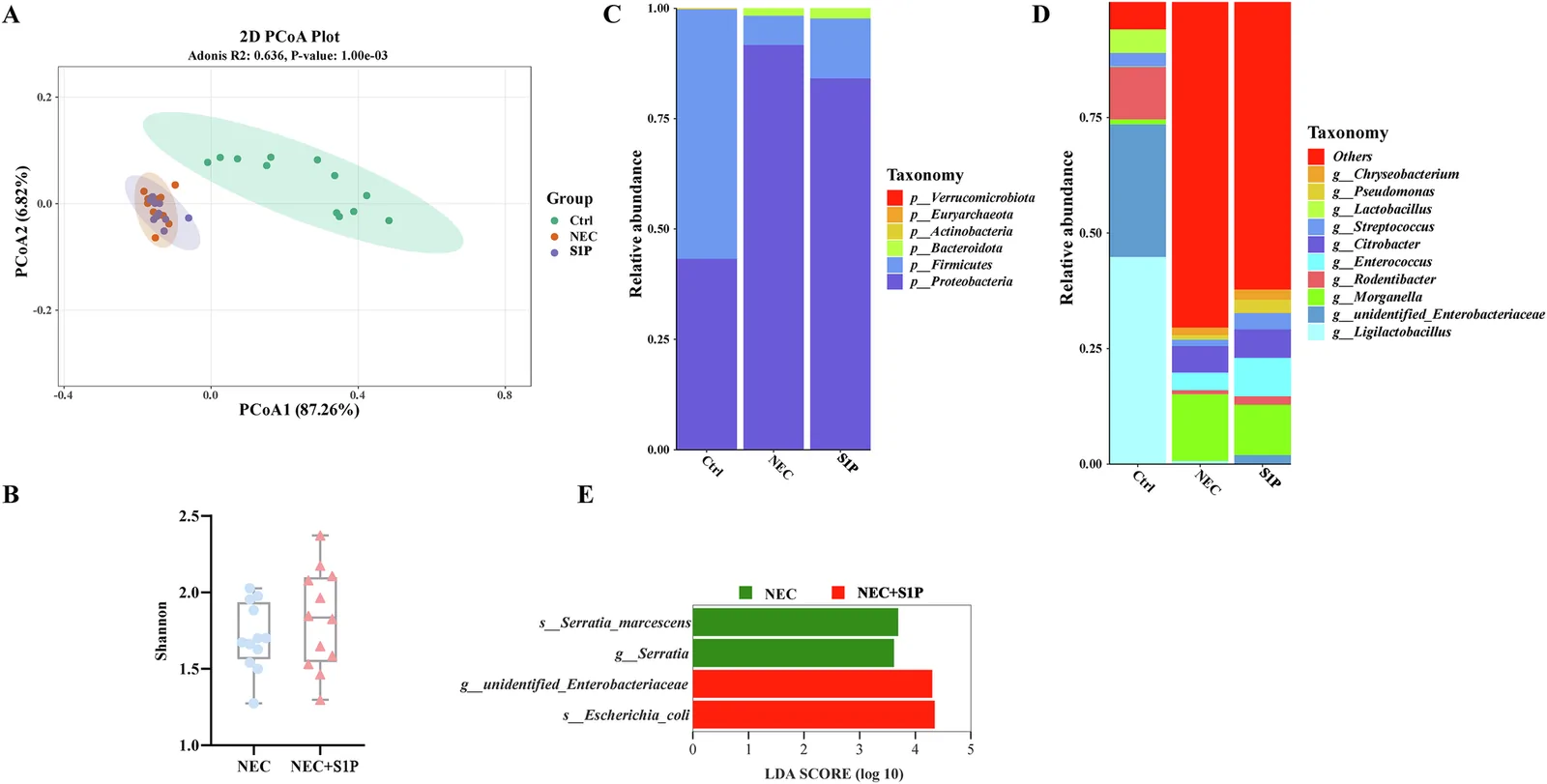
Sphingosine-1-phosphate (S1P) administration ameliorates gut microbiota structure in NEC mice. **A** Principal coordinates analysis (PCoA) of fecal microbiota 16S rRNA in mice. **B** Shannon diversity index of fecal microbiota. Microbiota distributions at the phylum (**C**) and genus (**D**) levels in mouse feces. **E** LEfSe analysis of mouse fecal microbiota.

It has been well-documented that active metabolites can influence host intestinal immunity and the gut barrier by modulating the host gut microbiota [8]. We next investigated whether S1P confers protection against NEC by modulating the gut microbial community. Using 16S rRNA gene sequencing, we analyzed the intestinal microbiota profiles of NEC mice with or without S1P intervention. There was no statistically significant difference in Shannon diversity index between the S1P and NEC groups, and principal coordinates analysis (PCoA) indicated that the two groups clustered closely together (Fig. 5A, B). At the phylum and genus levels, S1P treatment partially restored the microbiota dysbiosis induced by NEC, bringing the microbial composition closer to that of the healthy controls (Fig. 5C, D). LEfSe analysis further revealed that S1P intervention significantly reduced the relative abundance of *Serratia* (*g__Serratia*) and *Serratia marcescens* (*s__Serratia marcescens*), while increasing the abundance of *unidentified Enterobacteriaceae* (*g__unidentified Enterobacteriaceae*) and *Escherichia coli* (*s__Escherichia coli*) (Fig. 5E). These findings suggest that S1P may exert its protective effects not by enhancing overall microbial diversity, but rather through selective remodeling of the intestinal microbiota composition to mitigate NEC-associated dysbiosis.

### S1P protects IEC-6 cells from LPS-induced injury

**Fig. 6:**
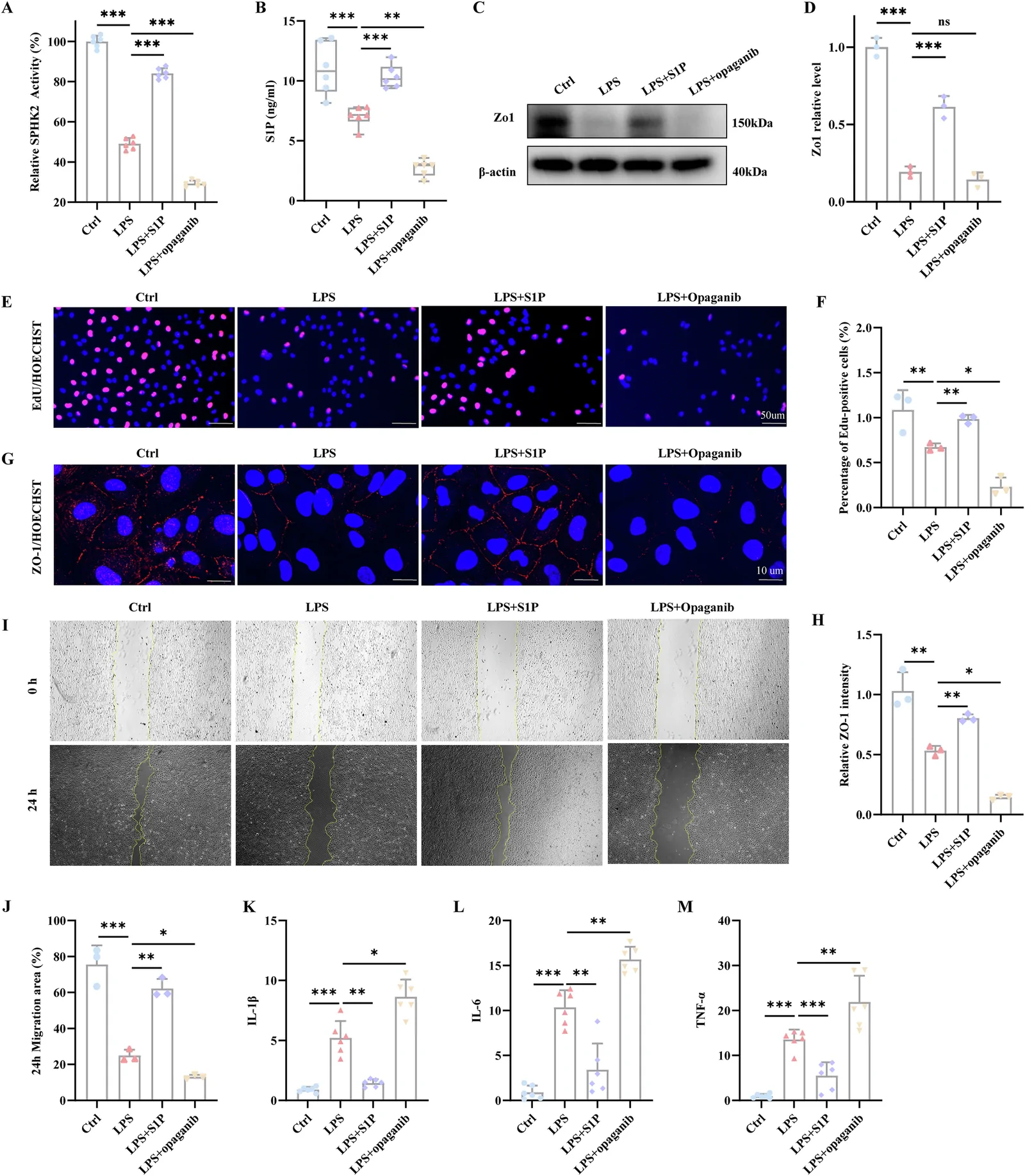
Sphingosine-1-phosphate (S1P) improves lipopolysaccharide (LPS)-induced proliferation, migration, barrier dysfunction, and inflammation in IEC-6 cells. **A** Sphk2 enzyme activity assay in IEC-6 cells. **B** S1P expression in IEC-6 cells. **C**, **D** Western blot analysis and quantification of ZO-1. **E**, **F** EDU staining and quantification of IEC-6 cell proliferation. **G**, **H** ZO-1 staining and quantification in IEC-6 cells. **I**, **J** Scratch wound healing assay and quantification of IEC-6 cell migration. RT-qPCR analysis of pro-inflammatory cytokine (interleukin [IL]-1β (**K**), IL-6 (**L**), and tumor necrosis factor [TNF]-α (**M**)) levels in IEC-6 cells. *P < 0.05, **P < 0.01, ***P < 0.001.

To explore the potential mechanisms underlying the Sphk2–S1P axis, we established an in vitro model by treating LPS-stimulated IEC-6 cells with exogenous S1P and opaganib. Sphk2 activity was reduced by LPS and was partially restored by S1P, whereas opaganib further exacerbated this reduction (Fig. 6A). ELISA analyses confirmed significantly reduced S1P levels following LPS stimulation, which were further decreased upon Opaganib (*P* < 0.05) (Fig. 6B). Western blot showed that S1P significantly restored the LPS-induced disruption of the barrier protein ZO-1, whereas opaganib markedly exacerbated this impairment (Fig. 6C, D). Markedly fewer EdU-positive cells after LPS treatment, with an even greater reduction observed following Opaganib. However, adding exogenous S1P substantially restored cell proliferation (Fig. 6E, F). IF staining revealed that S1P administration markedly enhanced the expression of ZO-1, whereas Opaganib exacerbated LPS-induced ZO-1 downregulation (Fig. 6G, H). Wound healing assays further confirmed that S1P effectively restored cell migration impaired by LPS, while Opaganib significantly suppressed migratory capacity (Fig. 6I, J). RT-qPCR showed significant upregulation of the pro-inflammatory cytokines in the LPS group, with further elevation observed in the Opaganib group. Notably, S1P intervention reversed this pro-inflammatory response (Fig. 6K–M). Collectively, these findings suggest that S1P may attenuate LPS-induced epithelial injury in vitro by modulating cell proliferation, migration, and inflammatory responses.

### S1P ameliorates LPS-induced mitochondrial dysfunction and redox imbalance in IEC-6 cells

**Fig. 7:**
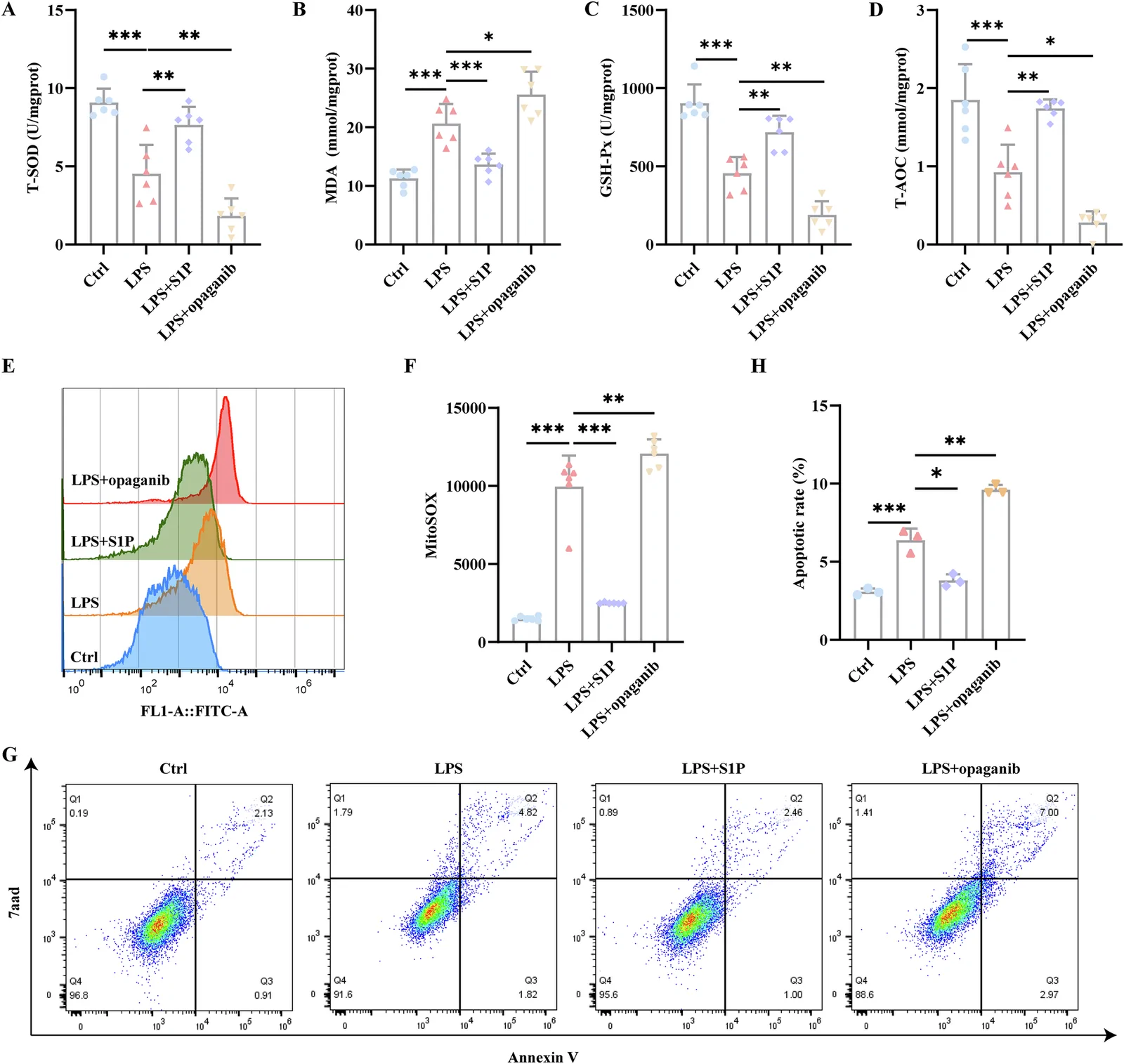
Sphingosine-1-phosphate (S1P) mitigates LPS-induced disruption of redox homeostasis and apoptosis in LPS-induced IEC-6 cells. Analysis of redox-related markers (total superoxide dismutase [T-SOD] (**A**), malondialdehyde [MDA] (**B**), glutathione peroxidase [GSH-Px] (**C**), and total antioxidant capacity [T-AOC] (**D**)) in IEC-6 cells. **E**, **F** Flow cytometry analysis and quantification of reactive oxygen species (ROS) levels in IEC-6 cells. **G**, **H** Flow cytometry analysis and quantification of apoptosis in IEC-6 cells.

To explore the potential involvement of S1P in mitochondrial function and oxidative stress, we performed a series of assays on LPS-stimulated IEC-6 cells. ELISA and ROS flow cytometry revealed that S1P significantly improved redox status, as evidenced by increased T-SOD, GSH-Px, and T-AOC levels, alongside decreased MDA levels. In contrast, Opaganib exerted the opposite effects (Fig. 7A–D). ROS quantification by flow cytometry confirmed that S1P markedly attenuated LPS-induced ROS accumulation, while Opaganib elevated ROS levels (Fig. 7E, F). LPS markedly increased apoptosis rates, which were further elevated upon Opaganib. However, S1P treatment significantly attenuated LPS-induced apoptosis (Fig. 7G, H).

**Fig. 8:**
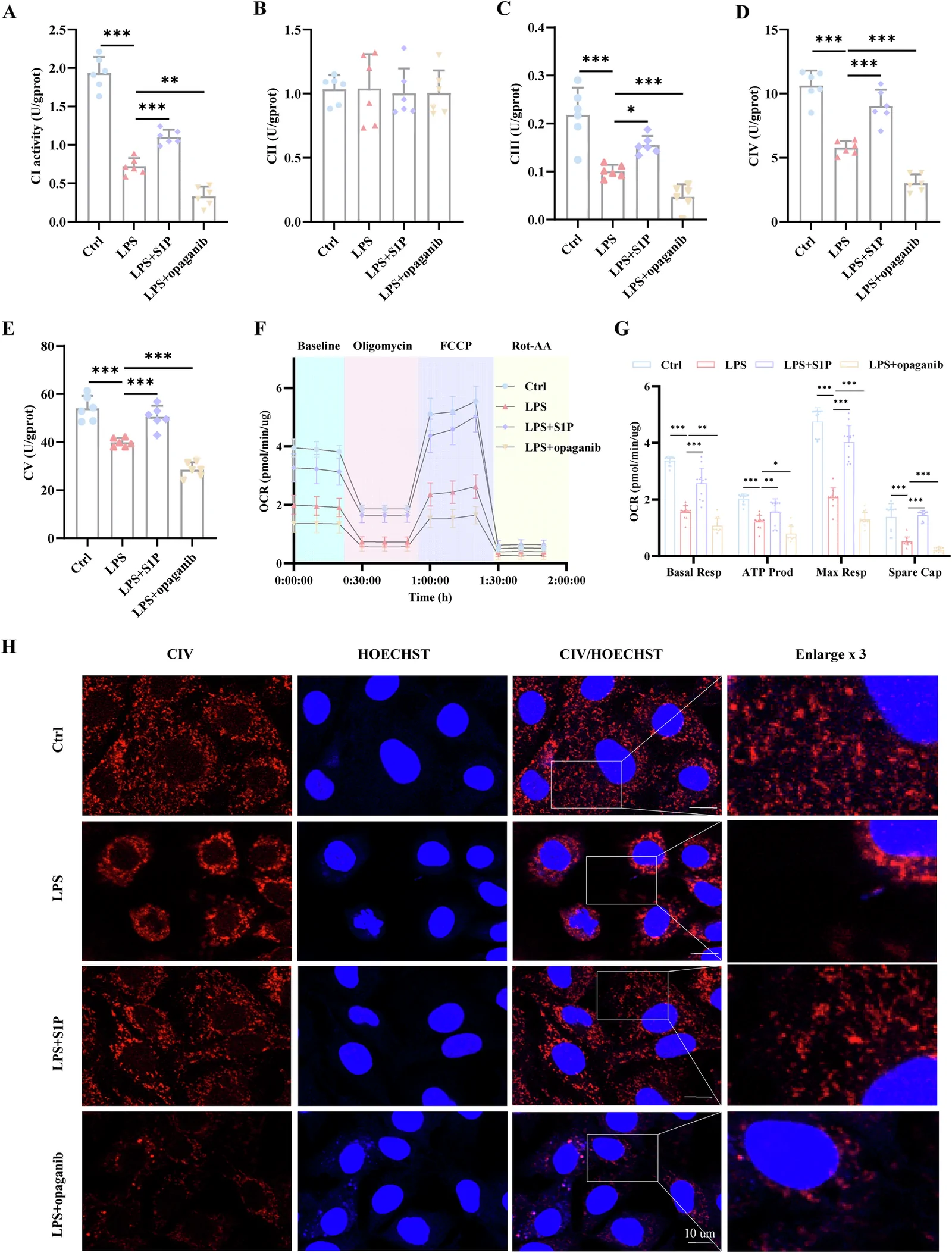
Sphingosine-1-phosphate (S1P) improves mitochondrial oxidative respiration in LPS-induced IEC-6 cells. Colorimetric assay measuring the expression of key mitochondrial respiratory chain enzymes (CI (**A**), CII (**B**), CIII (**C**), CIV (**D**), CV (**E**) in IEC-6 cells. **F** Mitochondrial respiratory function oxygen consumption rate (OCR) in IEC-6 cells. **G** Quantitative analysis and statistical chart of OCR values in IEC-6 cells. **H** Immunofluorescence staining of Complex IV (CIV) expression in IEC-6 cells. *P < 0.05, **P < 0.01, ***P < 0.001.

Previous studies have demonstrated that S1P attenuates ROS production by enhancing the efficiency of mitochondrial electron transport during oxidative phosphorylation, thereby limiting electron leakage and oxidative stress [15]. Consistent with this mechanism, our results showed that LPS stimulation markedly suppressed the activities of mitochondrial respiratory chain complexes I, III, IV, and V in IEC-6 cells, indicating profound impairment of mitochondrial oxidative phosphorylation. Notably, S1P treatment significantly restored the activity of these respiratory complexes, whereas inhibition of sphingosine kinase by Opaganib further exacerbated mitochondrial dysfunction (Fig. 8A–E). Seahorse XF metabolic flux analysis demonstrated that S1P significantly restored LPS-impaired mitochondrial energy metabolism, while Opaganib further aggravated mitochondrial dysfunction (Fig. 8F, G). IF staining consistently revealed reduced CIV expression in LPS-treated cells, which was reversed by S1P supplementation. Conversely, Opaganib led to further downregulation of CIV expression (Fig. 8H).

Collectively, these findings suggest that S1P may attenuate oxidative stress in LPS-stimulated IEC-6 cells, potentially involving mitochondrial-related processes.

### S1P reverses LPS-induced IEC-6 injury via S1PR1 signaling

**Fig. 9:**
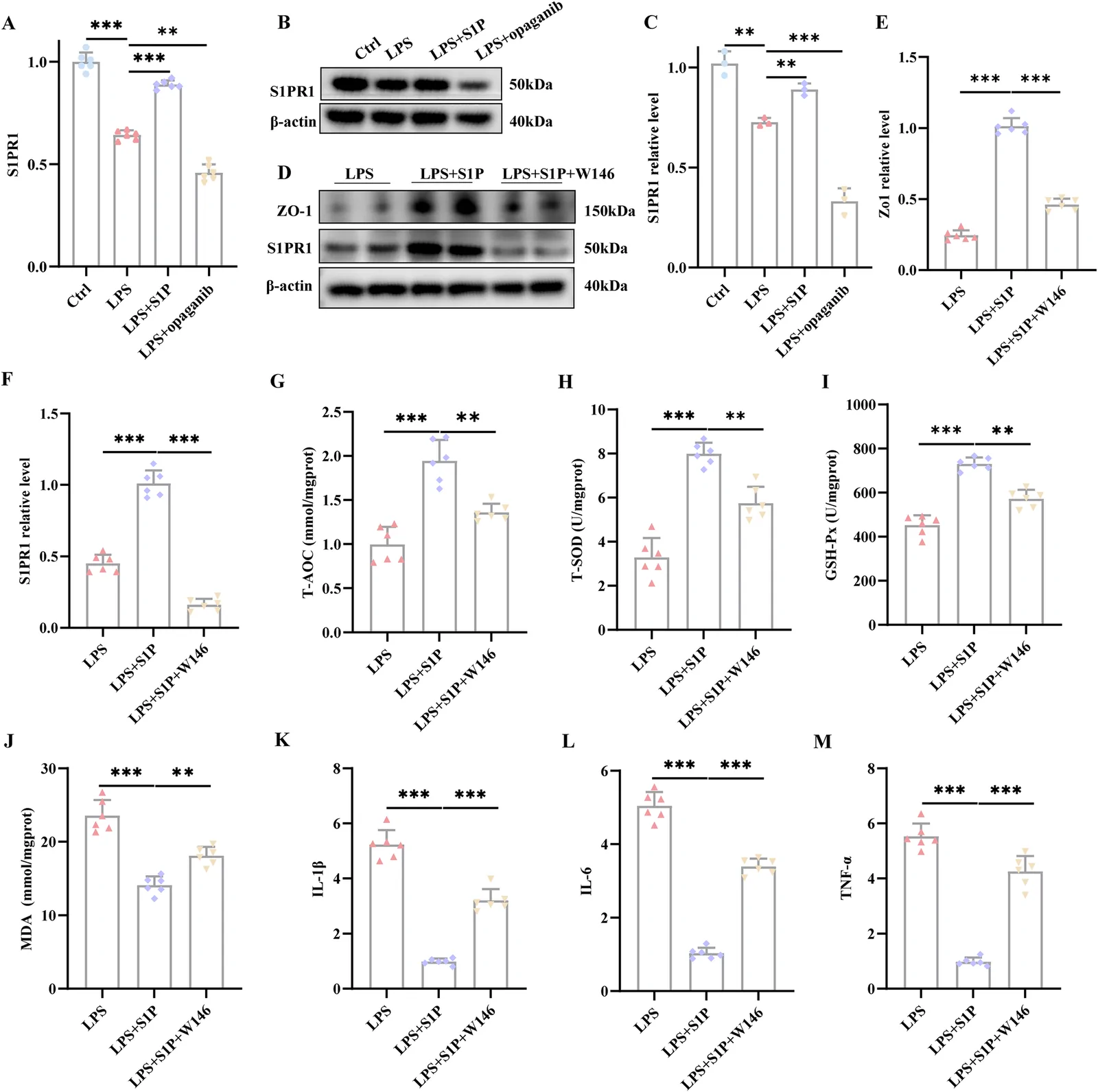
Sphingosine-1-phosphate (S1P) protects IEC-6 cells from LPS-induced injury via S1PR1 signaling. **A** qPCR analysis of S1PR1 expression. **B**, **C** Western blot analysis of S1PR1 expression. **D**–**F** Effects of S1PR1 antagonist W146 on S1PR1 expression and ZO-1 levels. ELISA analysis of redox-related markers (total antioxidant capacity [T-AOC] (**G**), total superoxide dismutase [T-SOD] (**H**), glutathione peroxidase [GSH-Px] (**I**), and malondialdehyde [MDA] (**J**)). RT-qPCR analysis of pro-inflammatory cytokine (interleukin [IL]-1β (**K**), IL-6 (**L**), and tumor necrosis factor [TNF]-α (**M**)) levels. **P < 0.01, ***P < 0.001.

To further determine whether S1P ameliorates LPS-induced IEC-6 injury via canonical receptor signaling, we assessed receptor expression by qPCR and Western blot. S1P significantly reversed LPS-induced downregulation of S1PR1, whereas opaganib further exacerbated this reduction (Fig. 9A–C). In contrast, S1PR2 and S1PR3 expression remained unchanged (Fig. S5). Moreover, blockade of S1PR1 with W146 markedly attenuated the protective effect of S1P against LPS-induced injury in IEC-6 cells. Western blot analysis further demonstrated that W146 significantly reduced S1PR1 expression in IEC-6 cells and abrogated the protective effect of S1P on the barrier protein ZO-1 under LPS stimulation (Fig. 9D–F). ELISA analysis showed that W146 significantly attenuated the protective effect of S1P against LPS-induced redox imbalance in IEC-6 cells (Fig. 9G–J). Consistently, qPCR analysis demonstrated that W146 markedly impaired the anti-inflammatory effect of S1P in LPS-treated IEC-6 cells (Fig. 9K–M).

### S1P modulates key transcriptional networks linked to oxidative stress and mitochondrial function

**Fig. 10:**
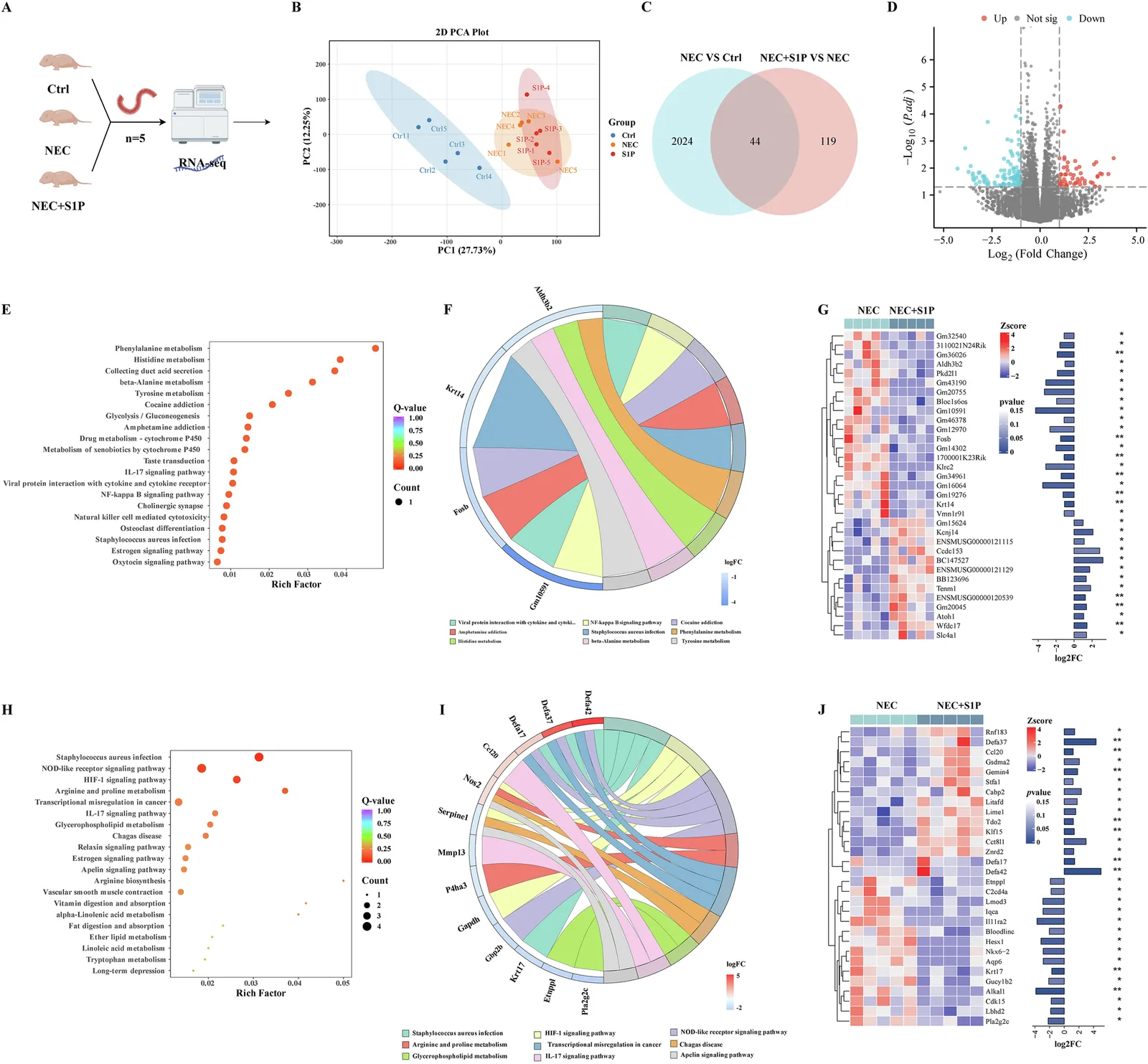
Transcriptomic sequencing analysis of mouse intestinal tissues following sphingosine-1-phosphate (S1P) gavage intervention. **A** Schematic representation of transcriptomic sequencing data from mouse intestinal tissues. Created with Figdraw (figdraw.com). **B** Principal component analysis (PCA) of transcriptomic sequencing data from mouse intestinal tissues. Venn diagram (**C**) and volcano plot (**D**) of differentially expressed genes (DEGs). Kyoto Encyclopedia of Genes and Genomes (KEGG) (**E**) and Gene Ontology (GO) (**F**) enrichment analysis of the intersecting DEGs between groups, with heatmap visualization (**G**). KEGG (**H**) and GO (**I**) enrichment analysis of S1P-regulated independent DEGs, with heatmap visualization (**J**). *P < 0.05, **P < 0.01.

To further explore the transcriptional regulatory effects of S1P in NEC, RNA-seq analysis was conducted on intestinal tissues from control, NEC, and S1P-treated NEC mice (Fig. 10A). PCA revealed distinct separation among the three groups along the PC1 and PC2 axes (Fig. 10B). Differential expression analysis identified 2068 differentially expressed genes (DEGs) between the NEC and control groups, while 163 DEGs (59 upregulated and 104 downregulated) were identified between the S1P-treated and NEC groups. Venn diagram analysis revealed 44 overlapping DEGs (16 upregulated, 28 downregulated) common to both comparisons (Fig. 10C, D). KEGG pathway enrichment analysis of the intersecting genes indicated significant enrichment in amino acid metabolism pathways, including phenylalanine metabolism and histidine metabolism, as well as glycolysis. These genes were also involved in immune-related pathways, including the IL-17 and nuclear factor kappa-light-chain-enhancer of activated B cells (NF-κB signaling pathways (Fig. 10E–G)).

Genes that did not differ significantly between the NEC and control groups but were independently regulated by S1P were identified. Functional enrichment analysis of these S1P-specific DEGs, excluding overlapping DEGs, revealed their involvement in pathways related to infection, the NOD-like receptor signaling pathway, and the hypoxia-inducible factor 1 (HIF-1) signaling pathway. Metabolically, these genes were enriched in arginine and proline metabolism, glycerophospholipid metabolism, tryptophan metabolism, and arginine biosynthesis (Fig. 10H–J). These pathways and genes are directly or indirectly involved in regulating oxidative stress responses and mitochondrial function.

These findings provide important mechanistic evidence that S1P may attenuate NEC by improving mitochondrial oxidative respiration and maintaining redox homeostasis in IECs.

### Bacteroides thetaiotaomicron reshapes intestinal sphingolipid metabolism and restores S1P in NEC

**Fig. 11:**
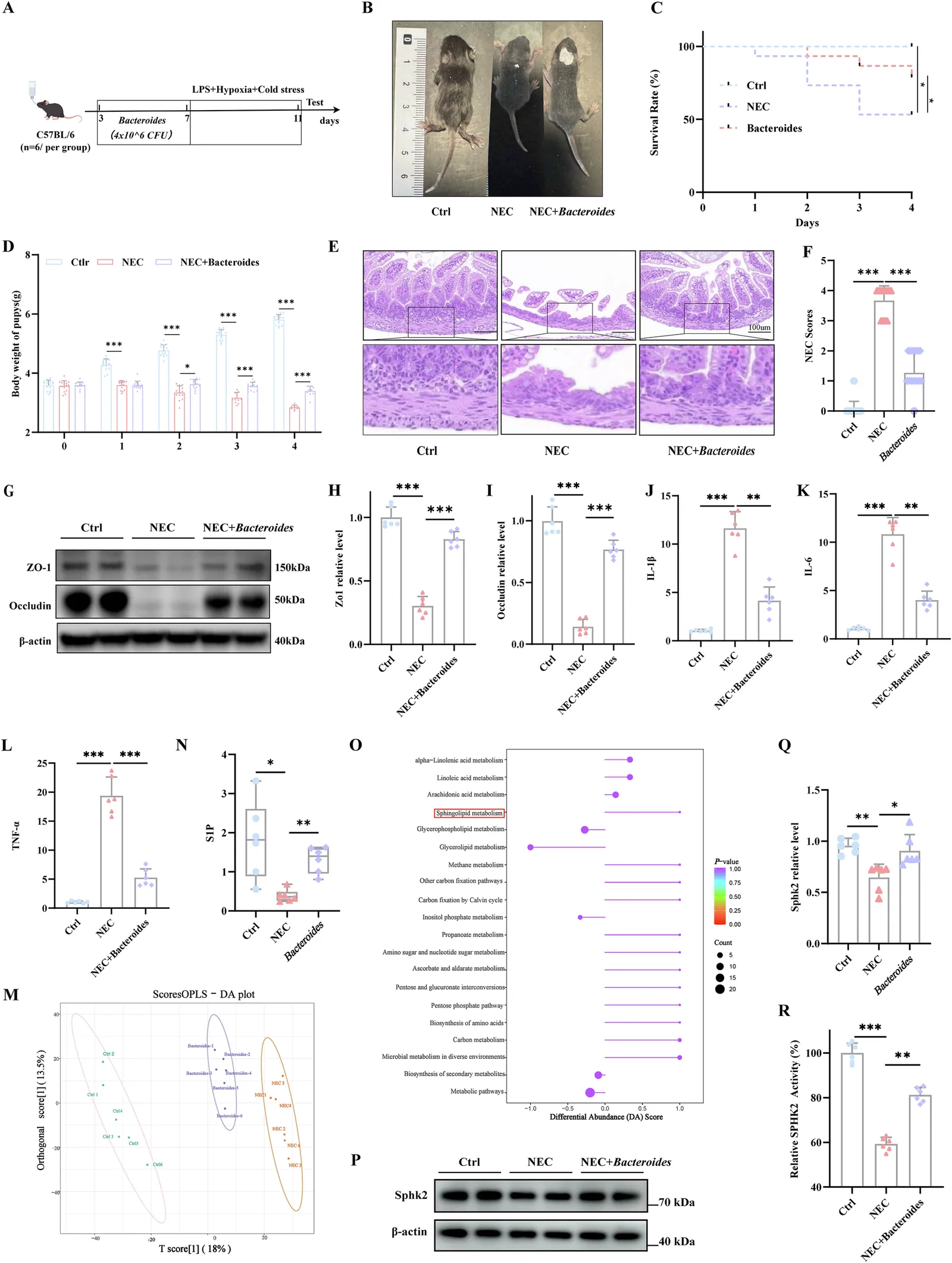
Sphingosine-1-phosphate (S1P) may be associated with the colonization abundance of *Bacteroides thetaiotaomicron.* **A** Intervention with *B. thetaiotaomicron* gavage. Created with Figdraw (figdraw.com). Representative images (**B**), survival curves (**C**), and body weight changes (**D**) of mice. Hematoxylin and eosin staining (**E**) and histopathological scores of distal ilial sections (**F**). **G**–**I** Western blot analysis and quantification of the intestinal barrier proteins ZO-1 and Occludin. RT-qPCR analysis of pro-inflammatory cytokines (interleukin [IL]-1β (**J**), IL-6 (**K**), tumor necrosis factor alpha [TNF-α] (**L**)) expression. **M** Orthogonal partial least squares-discriminant analysis (OPLS-DA) of non-targeted metabolomics in mouse intestinal tissues. **N** The expression of S1P in the three groups of intestinal tissues. **O** Kyoto Encyclopedia of Genes and Genomes (KEGG) pathway map of differential metabolites. **P**, **Q** Western blot analysis and quantification of Sphk2 expression. **R** Sphk2 enzyme activity assay. *P < 0.05, **P < 0.01, ***P < 0.001.

Reduced abundance of *Bacteroides* has been consistently reported as a characteristic feature of NEC-associated intestinal dysbiosis [16]. As a representative commensal species within the *Bacteroides* genus, *Bacteroides thetaiotaomicron* has been reported to contribute to intestinal barrier homeostasis by modulating host sphingolipid metabolism [17]. Accordingly, we administered *B. thetaiotaomicron* to NEC mice by oral gavage (Fig. 11A). *B. thetaiotaomicron* treatment significantly attenuated NEC (Fig. 11B–F). *B. thetaiotaomicron* significantly restored the NEC-induced loss of intestinal barrier proteins ZO-1 and Occludin (Fig. 11G–I) and reduced the levels of inflammatory cytokines (Fig. 11J–L). Untargeted metabolomics showed that *B. thetaiotaomicron* broadly mitigated NEC-induced metabolic disruption, with a prominent normalization of sphingolipid-associated features; notably, intestinal S1P was restored relative to the NEC group (Fig. 11M–O). In parallel, *B. thetaiotaomicron* increased the expression and enzymatic activity of intestinal Sphk2 (Fig. 11P–R).

Together, these data link *B. thetaiotaomicron*-mediated protection in NEC to a coordinated correction of intestinal sphingolipid metabolism, including recovery of S1P and induction of Sphk2.

## Discussion

**Fig. 12:**
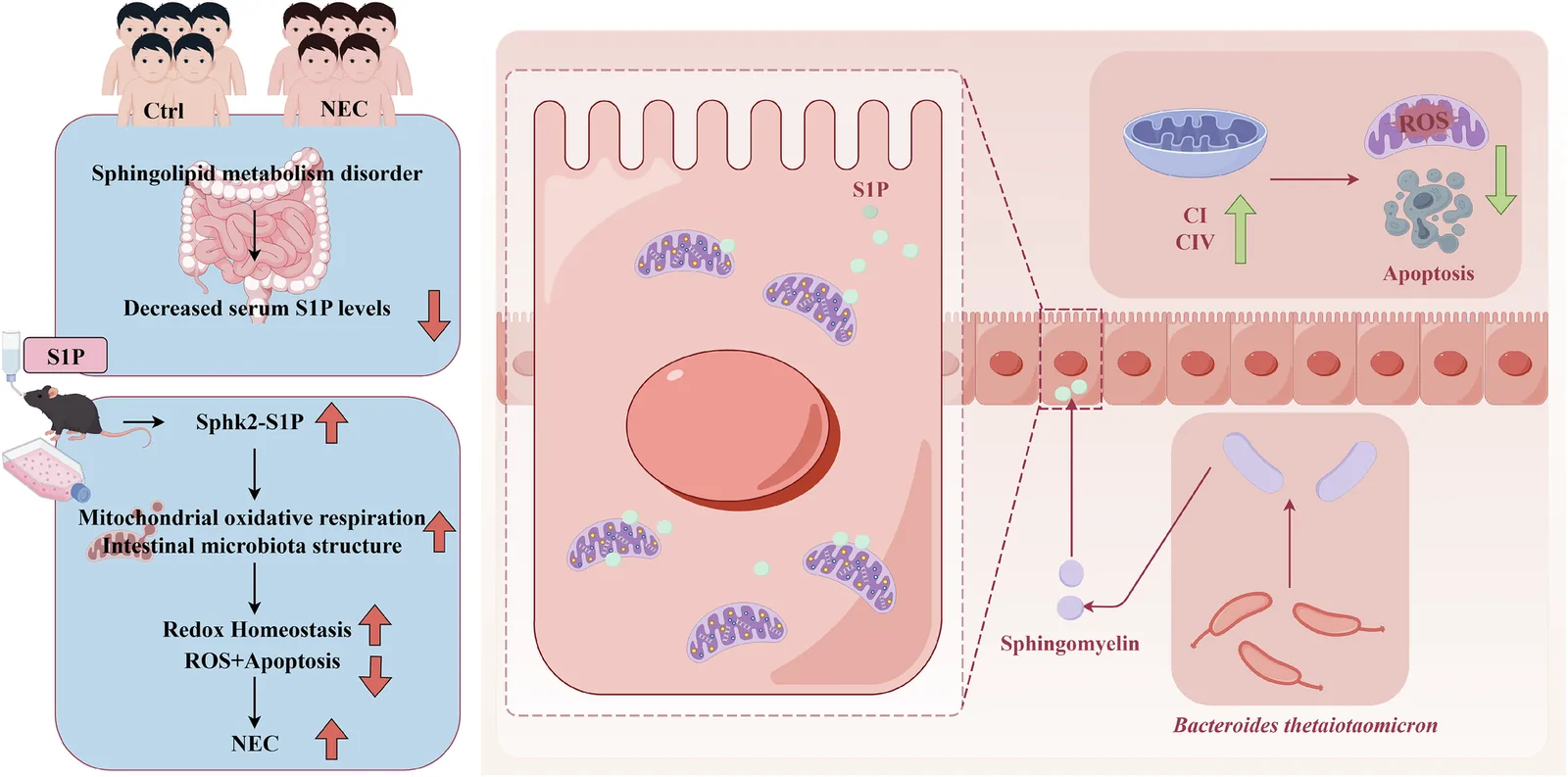
Sphingosine-1-phosphate (S1P) reshapes the gut microbiota, regulates mitochondrial oxidative respiration, improves intestinal epithelial redox homeostasis, reduces ROS and apoptosis, thereby protecting against necrotizing enterocolitis (NEC). Created with Figdraw (figdraw.com).

This study explored the potential role of S1P in NEC, a severe inflammatory disease of the premature intestine (Fig. 12). Our key findings included: (1) reduced serum S1P levels were associated with NEC occurrence, while exogenous S1P supplementation partially alleviated NEC-related phenotypes in experimental models; (2) S1P may be involved in mitochondrial redox regulation, which could be related to S1PR1 activation; (3) S1P was associated with changes in gut microbial composition; and (4) S1P levels showed a potential association with the abundance of B. thetaiotaomicron.

S1P is a bioactive sphingolipid with well-established roles in immune activation and cellular trafficking [12]. Growing evidence also implicates S1P is a key regulator of epithelial barrier function in mammalian cells [18–20]. However, its role in maintaining intestinal barrier integrity remains controversial, with studies reporting both pro-inflammatory and barrier-protective effects [21,22]. In the present study, we observed reduced plasma S1P levels in preterm infants with NEC, although some overlap between groups was present. Given the heterogeneity and complex pathophysiology of NEC, such variability is not unexpected. S1P levels were significantly associated with NEC, suggesting a potential link with disease-related processes. To further explore its functional relevance, we performed mechanistic studies in experimental NEC models. Inhibition of Sphk2 reduced S1P levels and aggravated NEC pathology, whereas exogenous S1P supplementation alleviated disease severity. In addition, S1P treatment partially restored gut microbial composition toward a healthier profile. Together, these findings suggest that S1P may play a role in NEC, potentially involving regulation of intestinal barrier function and microbial homeostasis.

Excessive oxidative stress accompanied by mitochondrial dysfunction is a hallmark feature of NEC [3,14]. Mitochondrial dysfunction leads to increased electron leakage from the electron transport chain and impaired antioxidant capacity, thereby directly triggering oxidative stress [23–25]. Previous studies have demonstrated that the Sphk2–S1P axis exerts beneficial effects on the mitochondrial redox environment [13]. We observed in the present study that suppressing Sphk2 expression significantly inhibited the activities of mitochondrial respiratory chain complexes I, III, IV, and V, leading to excessive ROS accumulation and elevated MDA levels, along with marked decreases in the expression of key antioxidant enzymes such as T-SOD and GSH-Px. S1P supplementation reversed these alterations. Further mechanistic analysis suggested that exogenous S1P exerts its effects in IEC-6 cells primarily through the S1P-S1PR1 signaling pathway. Intervention with the receptor-specific antagonist W146 demonstrated that blockade of S1PR1 partially attenuated the effects of S1P, consistent with previous reports indicating that S1PR1 regulates inflammatory responses via the PI3K/Akt pathway [26]. In addition, the Sphk2 inhibitor opaganib exacerbated NEC-related phenotypes in both in vivo and in vitro models, suggesting that under pathological stress, Sphk2 activity may contribute to maintaining endogenous S1P levels. Its inhibition may reduce available S1P, thereby impairing S1PR1-associated signaling. It should be noted that the effects of S1P may not be entirely dependent on receptor-mediated signaling. Exogenous S1P may also exert receptor-independent actions, such as intracellular uptake or direct regulation of mitochondrial function and other intracellular targets [27,28]. Importantly, S1P was significantly increased in IECs after gavage and was accompanied by elevated phosphoethanolamine, a well-established product of SGPL1-mediated irreversible S1P degradation. These findings provide evidence that exogenous S1P undergoes intracellular metabolic processing following epithelial uptake. Although our data support a direct protective role of exogenous S1P, they do not exclude the possibility that metabolites generated during intracellular S1P metabolism may also contribute to its protective effects. Future studies should further distinguish receptor-dependent from receptor-independent mechanisms using membrane-impermeable S1P analogs or endocytosis-blocking strategies, and further define the relative contributions of intracellular S1P metabolism and its downstream metabolites to the protective effects of exogenous S1P.

Our study reveals that intestinal S1P levels are gut microbiota-dependent, a finding entirely consistent with current cutting-edge understanding of how gut microbiota regulates sphingolipid metabolism [17,29]. It has been established that *B. thetaiotaomicron* ameliorates inflammatory bowel disease by regulating sphingolipid metabolism, thereby promoting intestinal barrier homeostasis [17]. We extend these findings by showing that *B. thetaiotaomicron* may serve as a therapeutic strain for NEC through restoration of microbiota-dependent S1P levels. This underscores its role not merely as a commensal reduced in disease, but as an active regulator of host-protective metabolic pathways, offering a mechanism-based strategy for NEC intervention via the gut microbiota–sphingolipid axis.

Although this study established a comprehensive framework from metabolomics discovery and model validation to signaling pathway analysis and identification of peripheral blood biomarkers in clinical samples, several limitations remain. First, despite demonstrating the association between reduced S1P levels and NEC onset, as well as its protective effects in experimental models, further studies are required before these findings can be translated into clinical metabolic interventions. Second, the mechanistic investigations were primarily based on LPS-stimulated IEC-6 cells, which represent a simplified inflammatory condition and may not fully recapitulate the complex pathophysiology of NEC; thus, validation in more physiologically relevant models is needed. In addition, although intracellular accumulation of exogenous S1P in IECs was directly demonstrated in the present study, stable isotope tracing was not performed. Therefore, the precise intracellular metabolic fate and flux of exogenous S1P remain to be determined. Future studies incorporating stable isotope tracing and cell-type-specific analyses will be important to further delineate the mechanisms of S1P metabolism and action. Moreover, the interaction between S1P and the gut microbiota remains to be further clarified, particularly how specific bacterial populations contribute to S1P production and regulation. Addressing these questions will provide a stronger foundation for future translational applications.

## Conclusion

This study provides a systematic analysis of the potential role of S1P in NEC pathogenesis. Our findings suggest that S1P may attenuate intestinal inflammation, epithelial apoptosis, and barrier dysfunction in experimental models. These effects may be associated with mitochondrial-related processes, redox homeostasis, and alterations in gut microbial composition, and could involve S1PR1-mediated signaling pathways. Furthermore, we observe that S1P levels may be associated with the abundance of *B. thetaiotaomicron*. Collectively, these results provide insights into S1P-related metabolic and signaling pathways in NEC and may support the future exploration of metabolism-based strategies, although further mechanistic and translational studies are warranted.

## Materials and methods

### Ethics statement

This prospective cohort study was approved by the Ethics Committee of the Children’s Hospital of Soochow University (Approval No. 2023CS130) and was conducted in accordance with the Declaration of Helsinki (1975, revised in 2008). Written informed consent was obtained from the guardians of all enrolled infants.

All animal experiments were conducted in strict accordance with institutional and national guidelines for the care and use of laboratory animals. Experimental protocols were approved by the Animal Experiment Ethics Committee of Soochow University (Approval No. SUDA20240911A37).

### Study participants and biological sample collection

This investigation included premature infants (<32 weeks of gestation) who were hospitalized in the Children’s Hospital of Soochow University and the Affiliated Suzhou Hospital of Nanjing Medical University between January 1, 2023, and June 30, 2024. Clinical data, laboratory test results, imaging data, and biological samples were collected during their hospitalization. The exclusion criteria included patients with severe congenital malformations, genetic metabolic disease, chromosomal abnormalities, or cases where the guardians refused participation in the study.

Serum and stool samples were collected within 24 h after birth and weekly until the infant was discharged. The biological specimens were aseptically obtained and promptly cryopreserved at −80 °C for future analytical procedures. The diagnostic criteria for NEC followed the International Practice Guideline Registry Platform (IPGRP)-2020CN004 guidelines.

### Reagents

Sphingosine 1-phosphate (S1P, S9666) was purchased from Sigma-Aldrich. Opaganib (T6750) was purchased from TargetMol (Shanghai, China). *Bacteroides thetaiotaomicron* (BNCC372688) was purchased from BeNa Culture Collection (Henan, China). The following primary antibodies were used: anti-zonula occludens-1 (ZO-1, 21773-1-AP, Proteintech, China; IF 1:500), anti-occludin (27260-1-AP, Proteintech, China; IF 1:500), anti-Ki67 (ab15580, Abcam, USA; IF 0.5–1 µg/mL), anti-sphingosine kinase 2 (SPHK2, 17096-1-AP, Proteintech, China; WB 1:2000; IP: 2.5 μg/mgprot), S1PR1 Polyclonal antibody (55133-1-AP, Proteintech, China; WB 1:1000), CoraLite® Plus 647-conjugated anti-cytochrome c oxidase subunit IV (COX IV, CL647-11242, Proteintech, China; IF 1:300), and anti-β-actin (66009-1-Ig, Proteintech, China; WB 1:20,000). The secondary antibodies included CoraLite®488-conjugated goat anti-rabbit immunoglobulin G (IgG, H + L) (SA00013-2), CoraLite®594-conjugated goat anti-rabbit IgG (H + L) (SA00013-4), and standard goat anti-rabbit IgG, all from Proteintech (China). Lipopolysaccharide (LPS), phosphate-buffered saline (PBS), and dimethyl sulfoxide (DMSO) were obtained from Solarbio (Beijing, China). Cell Counting Kit-8 (CCK-8) was purchased from Dojindo (Kumamoto, Japan). Hoechst 33342, 4′,6-diamidino-2-phenylindole (DAPI), and Protein A/G agarose magnetic beads were obtained from Beyotime (Shanghai, China).

### Commercial test kits

ELISA kits for sphingosine kinase 1 (SPHK1, CB14080-Mu) and SPHK2 (CB14748-Mu) were purchased from Cusabio Biotech (Shanghai, China), while the S1P ELISA kit (ELK9484) was obtained from ELK Biotechnology (Wuhan, China). Biochemical assay kits were acquired from Elabscience Biotechnology (Wuhan, China), including the Glutathione Peroxidase (GSH-Px) Activity Assay Kit (E-BC-K096-M0), Malondialdehyde (MDA) Colorimetric Assay Kit using the thiobarbituric acid (TBA) method (E-BC-K025-M), Myeloperoxidase (MPO) Activity Assay Kit (E-BC-K074-M), Total Superoxide Dismutase (T-SOD) Activity Assay Kit using the hydroxylamine method (E-BC-K019-M), and Total Antioxidant Capacity (T-AOC) Colorimetric Assay Kit (E-BC-K136-M). Mitochondrial function was assessed using the following kits from the same manufacturer: Complex I (NADH–CoQ Reductase, E-BC-K834-M), Complex II (E-BC-K835-M), Complex III (Coenzyme Q–Cytochrome C Reductase, E-BC-K836-M), Complex IV (Cytochrome C Oxidase, E-BC-K837-M), and Complex V (F₀F₁-ATPase/ATP Synthase, E-BC-K838-M). The BeyoClick™ EdU-488 Cell Proliferation Assay Kit (C0071S), ATP assay kit (S0026), and BCA protein assay kit (P0011) were obtained from Beyotime Biotechnology Co., Ltd. (Shanghai, China). The FITC Annexin V Apoptosis Detection Kit I (Pharmingen^TM^) was purchased from Becton, Dickinson and Company (Franklin Lakes, NJ, USA). The ADP-Glo^TM^ Kinase Assay Kit (V9101) was obtained from Promega Biotech Co., Ltd (Beijing, China). The CD326 (EpCAM) MicroBeads, mouse (130-105-958) were purchased from Miltenyi Biotec (Bergisch Gladbach, Germany). All assays were conducted according to the manufacturer’s instructions.

### Mice

C57BL/6 mice (6–8 weeks of age) were purchased from JOINN Laboratories (Suzhou, China) and housed in the Experimental Animal Center of the Children’s Hospital of Soochow University. All mice were maintained under specific pathogen-free conditions with controlled temperature, humidity, and a 12-h light/dark cycle. Each male was housed with two females in a single cage. On postnatal day 7 (P7), pups born on the same day were matched and randomly assigned to experimental groups for model establishment and subsequent interventions.

### Sex as a biological variable

Both male and female animals were examined in this study, and similar findings were reported for both sexes.

### Construction of the NEC animal model and related interventions

Newborn C57BL/6 mice were randomly assigned to experimental groups (*n* = 15 per group). Mice in the control group were naturally nursed by their dams. The NEC model was established using a widely validated method [30,31]. Between P7 and P10, the mice were fed a hyperosmolar formula consisting of a 2:1 mixture of Similac Advance infant formula (Abbott Nutrition, USA) and Esbilac puppy milk replacer (PetAg) via gavage every 6 h at a dose of 40 μL/g body weight. During days 2–4 of modeling, lipopolysaccharide (LPS, 4 μg/g) was added to the formula. Mice were subjected to a hypoxic environment (95% N₂ and 5% O₂) twice daily at fixed intervals for 10 min per session, followed by 10 min of cold exposure at 4 °C by placement in a refrigerated chamber.

The intervention groups included: S1P group (2 mg/kg, gavage, qd × 4 days), Opaganib group (5 mg/kg, gavage, qd × 4 days), and *B. thetaiotaomicron* (4 × 10^6^ CFU, gavage, qd × 4 days). During the experimental intervention period, mouse body weight changes and survival status were recorded daily. After the final treatment, the animals were euthanized, and their intestines were dissected to observe gross morphological changes. The distal ileal tissue was collected for histological evaluation, immunofluorescence staining, and ultrastructural examination. Effects of S1P on NEC mice and LPS-treated IEC-6 cells (see Fig. S6). Effects of Opaganib on NEC mice and LPS-treated IEC-6 cells (see Fig. S7). At the selected intervention doses, opaganib showed no apparent toxic or adverse effects in neonatal mice or IEC-6 cells.

To assess the local intestinal exposure of S1P following oral administration, intestinal tissues were collected at multiple time points for untargeted metabolomic analysis and S1P quantification. Sampling time points included T0 (pre-administration), T1 (30 min), T2 (1 h), T3 (3 h), T4 (6 h), and T5 (12 h), with three biological replicates per time point. In addition, intestinal tissue, liver, and blood samples were collected for S1P quantification. It should be noted that, due to technical limitations in obtaining luminal contents from neonatal mice, these were not analyzed separately in this study. During tissue collection, all intestinal samples were thoroughly rinsed with pre-chilled PBS (≥3 times) to minimize residual luminal contents. Based on intestinal S1P levels that peaked 30 min after gavage, this time point was selected to evaluate intracellular S1P and related metabolic alterations in IECs. Thirty minutes after S1P gavage, IECs were isolated from the mouse small intestine by EDTA dissociation and further enriched by EpCAM-based magnetic-activated cell sorting (MACS). To minimize interference from residual extracellular S1P, all isolation procedures were performed at 4 °C using pre-chilled PBS containing 2 mM EDTA, followed by extensive washing of EpCAM^+^ cells after MACS. Purified IECs were subsequently subjected to S1P ELISA and untargeted LC-MS/MS metabolomic analysis.

### Hematoxylin and eosin (H&E) staining

Distal ileal tissue was fixed for 24 h (4% paraformaldehyde), followed by ethanol gradient dehydration (70–100%) and clearing with xylene before paraffin embedding. Paraffin blocks were sectioned into continuous slices 3–5 μm in thickness using a Leica RM2235 microtome and mounted onto glass slides, which were then baked at 60 °C for 1 h. Next, the sections were dewaxed in xylene and rehydrated through an ethanol gradient, followed by staining with Harris hematoxylin for 5 min, differentiation in 1% acid alcohol, bluing with 0.1% ammonia water, and subsequent staining with 1% eosin Y for 1 min. The stained tissue sections underwent sequential dehydration using graded ethanol solutions, were transparentized in xylene, and finally coverslipped with neutral balsam. All samples were evaluated histologically by two independent pathologists blinded to the experimental groups, using a standardized scoring system based on established criteria [32].

### Immunofluorescence staining of mouse intestinal tissue

The tissue sections were first dewaxed in xylene (two 10-min washes) and progressively hydrated through an ethanol series (50–100%, 5 min per concentration) before PBS rinses (three 5-min cycles). To facilitate antigen retrieval, tissue sections were incubated in citrate buffer at 95–100 °C for 20 min, followed by cooling to ambient temperature and PBS washing. Primary antibody incubation proceeded overnight at 4 °C. Following PBS washes, tissue sections were incubated with species-matched Alexa Fluor-labeled secondary antibodies (1:500 dilution) for 1 h at RT in the dark. Nuclei were subsequently stained with DAPI (5 µg/mL) for 5 min. Images were acquired using an Olympus confocal microscope (NY, USA) with standardized settings (laser intensity, gain, and exposure duration). A minimum of three randomly selected fields per sample were analyzed, and fluorescence intensity was quantified using ImageJ software. Image analysis was conducted in a blinded manner by the observer.

### Transmission electron microscopy (TEM) analysis of mouse intestinal tissue

Freshly collected mouse ileum tissues were immediately fixed in 2.5% glutaraldehyde (0.1 M phosphate buffer, pH 7.4) at 4 °C for 24 h, followed by post-fixation with 1% osmium tetroxide at RT for 2 h. Following dehydration through a graded ethanol series (50–100%), tissues were infiltrated with propylene oxide and embedded in EPON 812 resin. Ultrathin sections (70–90 nm) were subsequently cut using a Leica UC7 ultramicrotome. The sections were examined using a Hitachi TEM system (Japan), and images were captured with an AMT XR41 CCD camera. For each sample, at least 10 random fields were analyzed, and the intestinal ultrastructure was examined.

### IEC-6 cell culture and Intervention

The IEC-6 rat IEC line (FuHeng Biology, Shanghai, China) was cultured in high-glucose DMEM (Gibco, USA) supplemented with 10% fetal bovine serum and 1% penicillin-streptomycin, and maintained at 37 °C in a humidified atmosphere containing 5% CO₂. The study was organized into four distinct groups: Control, LPS, LPS + S1P, and LPS + Opaganib. Briefly, 2 × 10⁵ cells were seeded in 6-well plates and incubated for 12 h to allow attachment. The cells were subsequently treated with LPS (20 μg/mL), S1P (0.1 μM), and Opaganib (25 μM) for 48 h before downstream assays.

To further determine whether the effects of S1P are mediated through the S1PR1 pathway, the selective S1PR1 antagonist W146 was introduced into the experimental system. IEC-6 cells were pretreated with W146 for 30 min prior to LPS stimulation, followed by incubation with the indicated treatments until the designated time point. The concentration of W146 was selected based on its commonly used effective range in vitro and cell viability assessment, and 10 μM was ultimately used for functional blockade (Fig. S8).

### Cell immunofluorescence staining

Cells (1 × 10⁵/well) were seeded in confocal dishes and transfected as previously described. After treatment, they were washed with PBS, fixed in 4% paraformaldehyde, and permeabilized with 1% Triton X-100 for 5 min. Blocking was performed with 5% goat serum at room temperature for 2 h, followed by overnight incubation at 4 °C with primary antibodies. Afterward, fluorescently labeled secondary antibodies were applied under light-protected conditions and incubated at 37 °C for 1 h. Images were acquired using an Olympus confocal microscope (NY, USA).

### Immunoprecipitation and Sphk2 Kinase Activity Assay

Protein lysates were incubated with a validated, specific anti-SPHK2 antibody at 4 °C for 2–4 h, followed by the addition of Protein A/G agarose magnetic beads and overnight rotation at 4 °C. Protease and phosphatase inhibitors were included in both the lysis and wash buffers to preserve enzyme activity. Immunoprecipitated proteins were washed three times to remove non-specifically bound proteins and subsequently collected for kinase activity measurement. Sphk2 kinase activity was measured using the ADP-Glo^TM^ Kinase Assay kit according to the manufacturer’s instructions. Luminescence signals were converted to ADP production using an ATP-ADP standard curve and normalized to the amount of Sphk2 protein in the immunoprecipitated samples. Absolute enzyme activity is expressed as pmol ADP/min/μg Sphk2. To account for potential variations in IP recovery, results in the main text are presented as relative kinase activity (% of control), with absolute activity data provided in the Supplementary Material (pmol ADP/min/μg Sphk2) (Fig. S9). Substrate concentrations and luminescence signals were confirmed to be within the linear range of the assay.

### Seahorse

Cellular energy metabolism was evaluated using a Seahorse XF Analyzer (Agilent Technologies, USA). IEC-6 (2.5 × 10⁴ cells/well) were plated on Seahorse XF-specific culture plates and incubated overnight for cell attachment. Prior to testing, the medium was replaced with Seahorse XF-specific assay medium, and the plates were incubated in a 37 °C, CO₂-free environment for 45–60 min to equilibrate temperature and pH. The sensor probe plate was hydrated overnight in Seahorse XF calibration solution at 37 °C in a CO₂-free incubator. Metabolic modulators were subsequently loaded into the designated injection ports. Following instrument calibration, the cell culture plate was placed into the analyzer, and either a mitochondrial or glycolytic stress test was performed. During the test, oxygen consumption rate (OCR) was monitored in real-time. Data were normalized to protein concentration, analyzed using Wave software 2.0 (Agilent Technologies), and exported to GraphPad Prism 10 for statistical analysis. The related parameters were calculated according to the manufacturer’s guidelines.

### Scratch wound assay

IEC-6 cells (2 × 10⁵ cells/well) were plated in 6-well plates and cultured for 12 h. Intervention with corresponding reagents, cells were grown to confluence. A standardized wound was generated using a 200 μL pipette tip, mimicking mechanical injury. After PBS washes to clear debris, experimental treatments were applied. Initial (0 h) and 24 h post-scratch images were acquired via inverted microscopy. Migration rates were quantified by measuring scratch width differences using ImageJ and calculating gap closure percentages.

### Cell viability assay

IEC-6 cells (8000/well) were seeded into 96-well plates (100 μL/well) and cultured for 24 h. The medium was then replaced with fresh medium containing group-specific treatments. After an additional 24-h incubation, CCK-8 reagent was diluted 1:10 in serum-free medium, and 100 μL of the working solution was added to each well. Absorbance at 450 nm was recorded after a 2-h incubation using a microplate reader. Cell viability was calculated based on absorbance and normalized to evaluate drug-induced effects on proliferation and cytotoxicity.

### Determination of gene expression in intestinal tissue by real-time quantitative polymerase chain reaction (RT-qPCR)

Intestinal tissue RNA was extracted using TRIzol reagent (Thermo Fisher Scientific, USA), and cDNA was synthesized from the RNA using a reverse-transcription kit as per the protocol. PCR analysis was conducted with SYBR Master Mix on a Roche LightCycler 480. β-Actin was used as the internal control, with mRNA levels quantified via the 2^−ΔΔCt^ method. Primer sequences are listed in Table S3.

### Western blotting

After treatment, cells or tissues were lysed in RIPA buffer (Beyotime, China) with protease and phosphatase inhibitors (Roche, Switzerland). The lysates were homogenized on ice, then centrifuged at 12,000 × *g* for 15 min at 4 °C to collect the supernatant. Protein concentration was quantified using a BCA assay kit (Thermo Fisher Scientific, USA) following the manufacturer’s protocol. Equal protein amounts were separated by SDS-PAGE and transferred to PVDF membranes (Millipore, USA). The membranes were blocked with 5% non-fat milk in TBST (0.1% Tween-20) for 1 h at room temperature, followed by overnight incubation with primary antibodies at 4 °C. After 1 h incubation with HRP-conjugated secondary antibodies at room temperature, signals were detected using ECL reagents (Bio-Rad, USA) and visualized with a Tanon 5200 imaging system (Tanon, China). Band intensities were quantified using ImageJ software. The original Western blots can be found in the Supplementary Material.

### Apoptosis assay by flow cytometry

Following the cellular experiments, PBS buffer (pH 7.4) was used to wash the cells twice before resuspension in the Apoptosis Detection Kit I’s staining solution. Subsequently, the cell suspensions were incubated with 5 μL each of Annexin V and 7-AAD for 15 min at ambient temperature. Flow cytometric analysis (DxFLEX, Beckman Coulter, USA) was performed within 60 min of staining.

### Reactive oxygen species

Upon establishing the cell model, enzymatic detachment (trypsin) was performed, followed by centrifugation (800 rpm, 5 min) to harvest cells. The pellets were treated with 10 μM DCFH-DA (Sigma-Aldrich) in darkness (37 °C, 20 min), with intermittent agitation. Post-incubation, PBS washes (×3) eliminated excess probe before resuspension in 300–400 μL PBS. Fluorescence was quantified via DxFLEX flow cytometry (minimum 10,000 events/sample), with unstained cells establishing baseline thresholds. H2O2-exposed cells served as positive controls. FlowJo software processed data (MFI ratio analysis).

### Untargeted metabolomics

For metabolite extraction from intestinal tissues, samples were homogenized in a pre-cooled 2:2:1 methanol:acetonitrile:water solvent containing internal isotopic standards. The homogenates were subjected to repeated shaking and ultrasonication (35 Hz for 4 min, followed by 5 min in an ice bath), processed three times, precipitated at −40 °C, and centrifuged at 12,000 rpm for 15 min at 4 °C. The resulting supernatants were collected, and a pooled quality control (QC) sample was prepared. For human serum metabolomics, 100 μL of serum was mixed with 400 μL of pre-cooled methanol:acetonitrile (1:1, v/v) containing internal isotopic standards to precipitate proteins. Samples were vortexed for 1 min, incubated at −20 °C for 1 h, and centrifuged at 12,000 rpm for 15 min at 4 °C. The supernatants were collected, and a pooled QC sample was generated. All samples were analyzed by LC-MS/MS using a HILIC column (2.1 × 100 mm, 1.7 μm). Gradient elution was performed with acetonitrile and 25 mM ammonium acetate-ammonia as the mobile phase. Data acquisition was conducted in both positive and negative ionization modes using an information-dependent acquisition strategy.

KEGG pathway enrichment analysis was performed using the MetaboAnalyst platform. The methodology strictly adhered to metabolomics standardization guidelines (COordination of Standards in MetabOlomicS [COSMOS]), with multi-dimensional QC to ensure data reliability.

### 16S rDNA gene analysis

*Wuhan Maiwei Metabolic Biotechnology Co., Ltd*. performed 16S rRNA sequencing on fecal samples (*n* = 12 per group). Upon collection, fecal samples were immediately frozen in liquid nitrogen. Total DNA was extracted using the sodium dodecyl sulfate method, and the V3–V4 region was amplified using barcode-labeled primers [341F (CCTAYGGRBGCACAG) and 806R (GGACTACNNGGGTATCTAAT)]. The amplicons were screened by 2% agarose gel electrophoresis, purified using magnetic beads, and equimolarly pooled to construct the Illumina NovaSeq6000 PE250 sequencing library. The raw data underwent QC analysis using fastp to filter out low-quality reads (*Q* < 15, length <150 bp, and sequences containing *N* bases). After merging paired-end reads using Fast Length Adjustment of SHort reads (FLASH) [33], operational taxonomic units were clustered at 97% similarity using the UPARSE algorithm [33]. Taxonomic annotation was performed based on the SILVA database (v138) with a confidence threshold of 0.8–1. Data were analyzed using QIIME2 [34] and the R platform. Alpha diversity was assessed using the Shannon index, and beta diversity was evaluated using UniFrac distance. Distinct microbial compositions across groups were determined by Linear discriminant analysis Effect Size (LEfSe) analysis (LDA score >3.5, *P* < 0.05).

### RNA sequencing (RNA-seq) and analysis

RNA-seq was conducted on intestinal tissues from each treatment group (*n* = 5/group). Total RNA isolation employed TRIzol reagent, followed by mRNA enrichment (RIN ≥ 7.0) via oligo(dT) beads. The purified mRNA was then fragmented into ~300 bp segments and reverse-transcribed into cDNA for library preparation. The cDNA was subjected to end repair, A-tail addition, and adapter ligation to prepare the sequencing library. Library concentrations were assessed using Qubit, and sequencing was subsequently conducted on the NovaSeq 6000 platform with paired-end 150 bp reads. Raw data quality was controlled with fastp (filtering out *Q* < 20, adapter sequences, and >5% *N* bases). Sequencing depth was ≥20 M reads/sample. The clean reads were then aligned to the mouse reference genome (GRCm38) using Hisat2 [35], and gene expression quantification was performed using RSEM [36]. Differential expression was analyzed with DESeq2 (|log2FC| ≥ 1, *P* < 0.05). GO and KEGG pathway enrichment analyses were conducted using the clusterProfiler package.

### Statistical analysis

All experiments were performed in triplicate. Statistical analysis was performed using R Studio and GraphPad Prism 10. Shapiro-Wilk and Levene’s tests assessed data normality and variance homogeneity, respectively. For comparisons between two groups, normally distributed data were analyzed using the Student’s *t*-test, while the Mann–Whitney *U* test was applied to non-parametric data. When comparing multiple groups, one-way ANOVA followed by Tukey’s post hoc test was used for data meeting parametric assumptions, whereas the Kruskal-Wallis test with Dunn’s correction was used otherwise. Correlations between variables were examined via Spearman’s rank method. Data visualization was carried out using the *ggplot2* package in R. Receiver operating characteristic (ROC) curve analysis was conducted with the *pROC* package to assess diagnostic accuracy. Survival differences were evaluated using Kaplan-Meier curves and compared with the log-rank test. Results are reported as mean ± SEM for normally distributed variables, or median with interquartile range for skewed data. Two-sided *P* < 0.05 was considered statistically significant.

## Supplementary information

Supporting Information

Western blot_raw bands

## Data Availability

The datasets used and/or analyzed in this study are available from the corresponding author on reasonable request.
