# Supplementary material for: Sphingosine-1-phosphate ameliorates necrotizing enterocolitis by regulating mitochondrial redox homeostasis and gut microbiota
Source: Cell Death Dis. 2026 Jul 31;17(1):913. doi: 10.1038/s41419-026-09144-8 (PMC13720040; doi:10.1038/s41419-026-09144-8)

Figure 3I

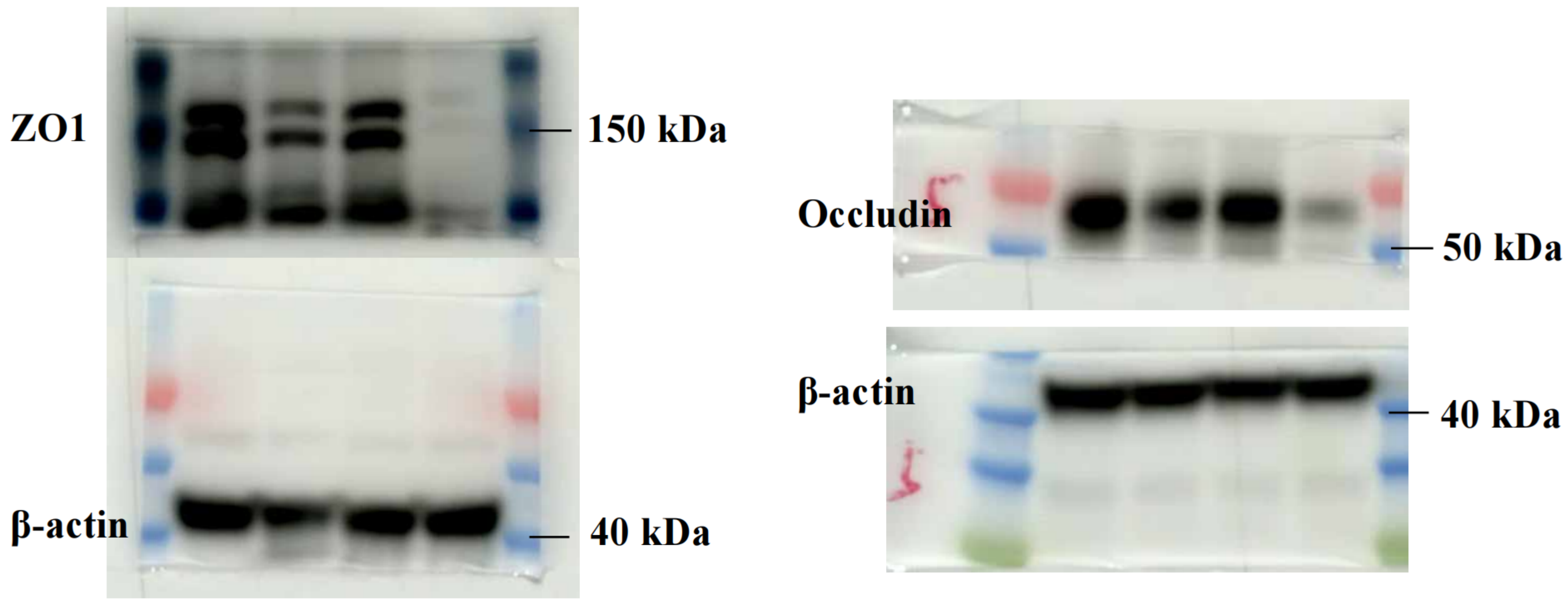

Figure 6C

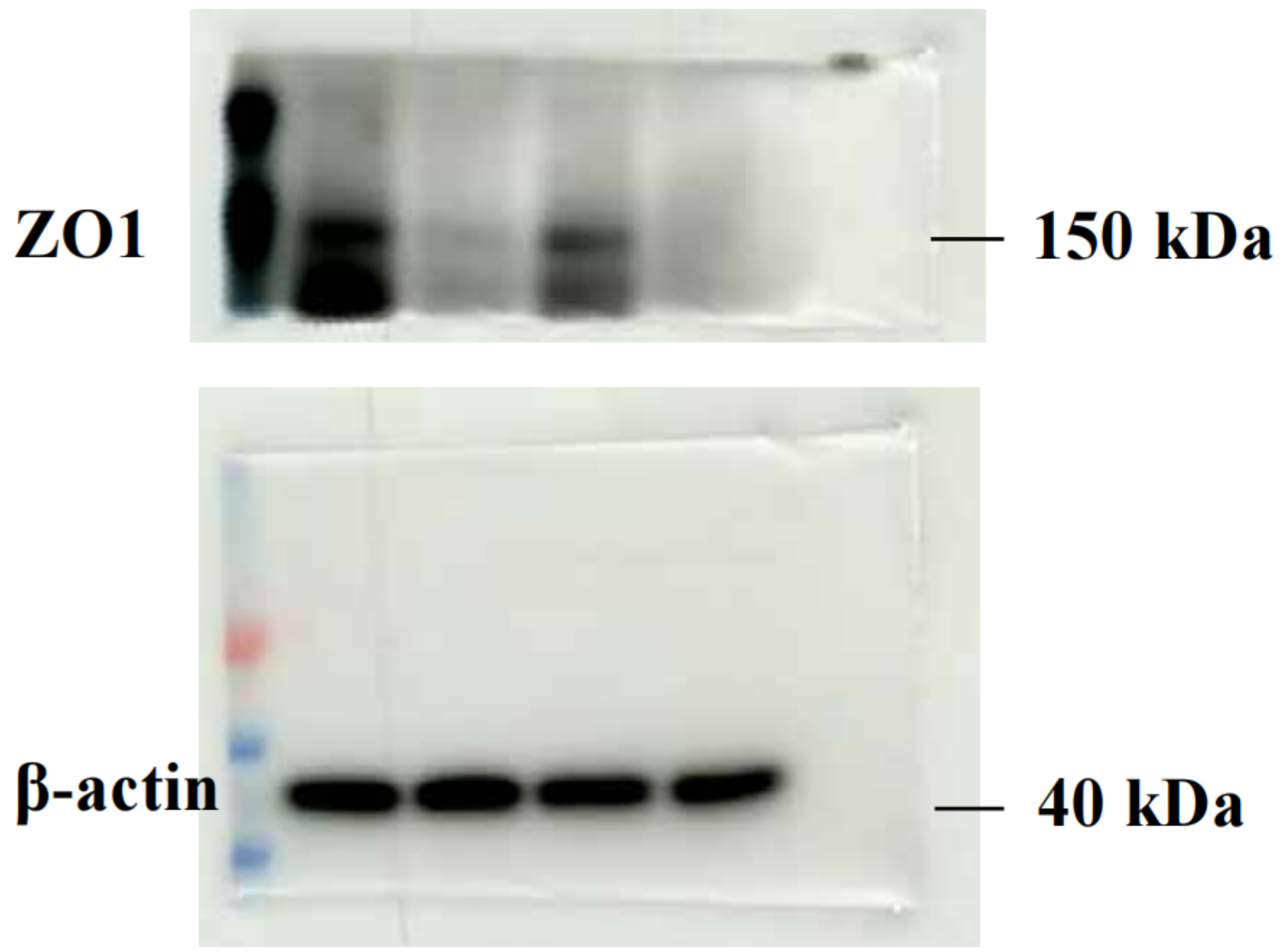

Figure 9B

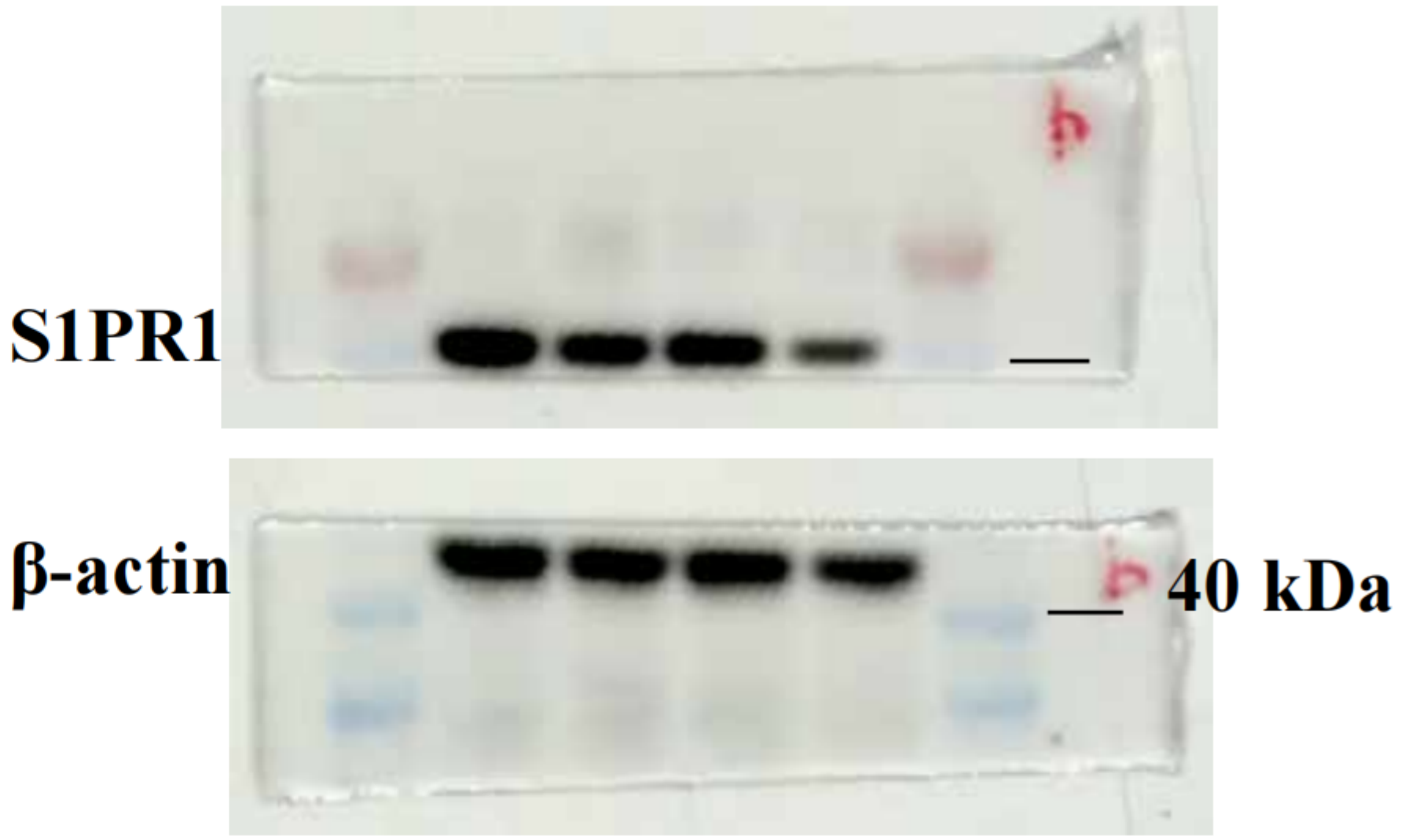

Figure 9D

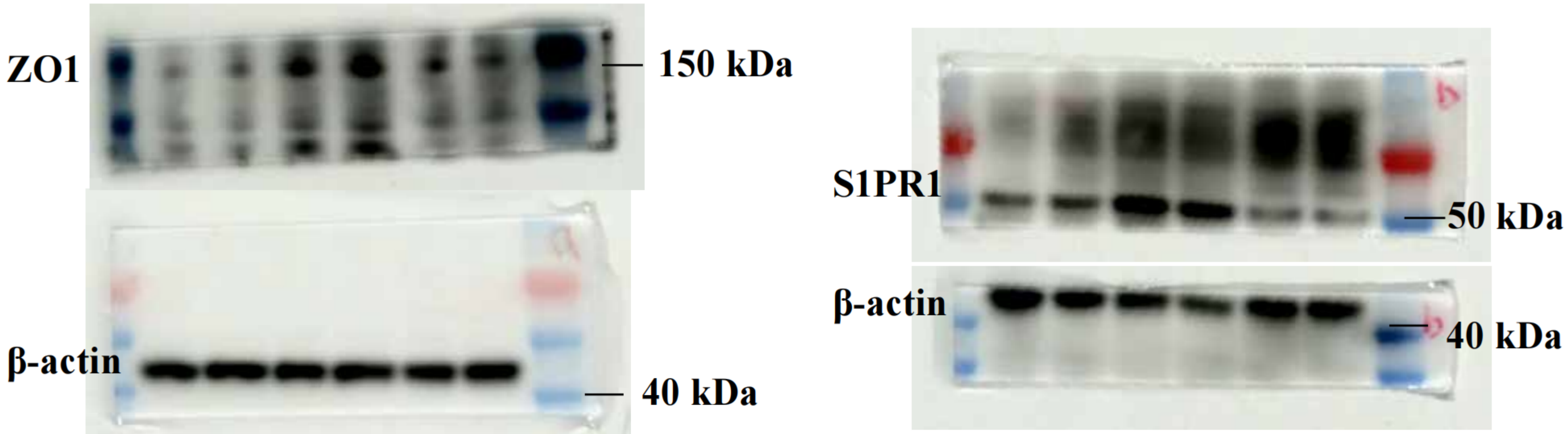

Figure 11G

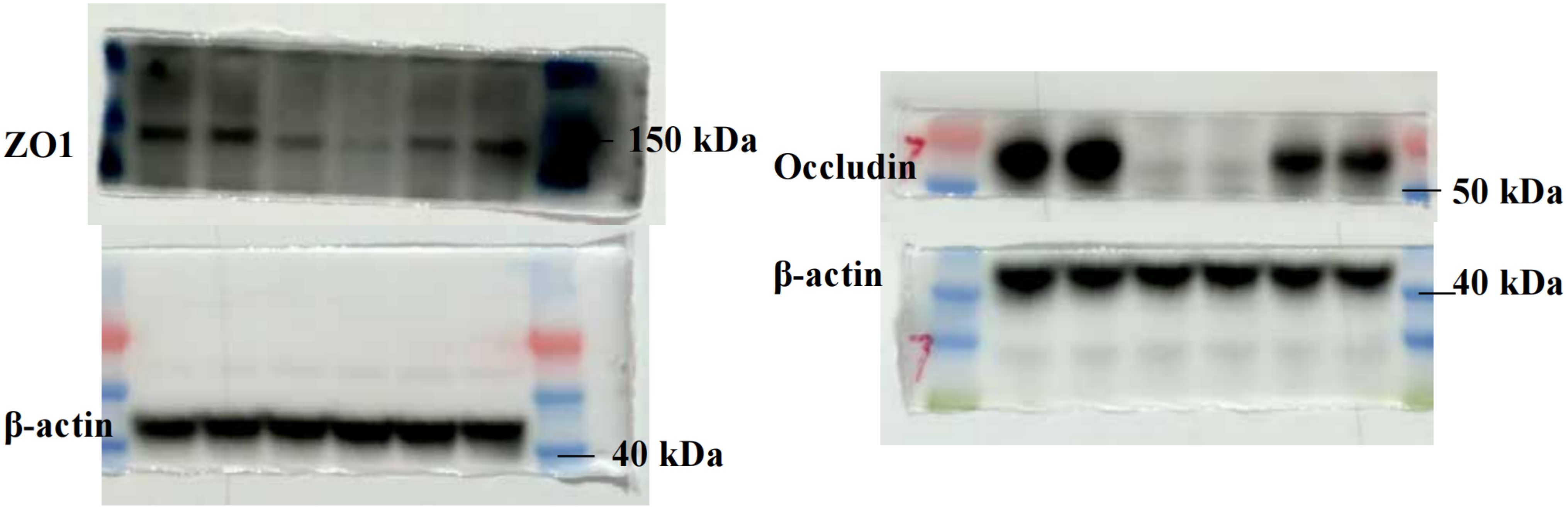

Figure 11P

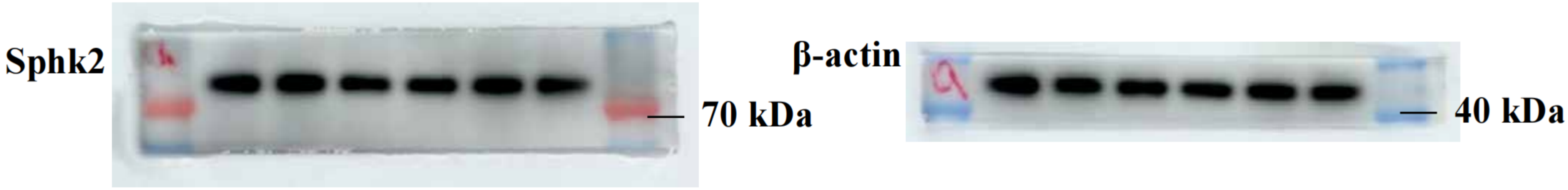

Supplement: Supplementary file 1 [file 41419_2026_9144_MOESM2_ESM.pdf]
