# Supplementary material for: Sphingosine-1-phosphate ameliorates necrotizing enterocolitis by regulating mitochondrial redox homeostasis and gut microbiota
Source: Cell Death Dis. 2026 Jul 31;17(1):913. doi: 10.1038/s41419-026-09144-8 (PMC13720040; doi:10.1038/s41419-026-09144-8)
Supplement: Supplementary file 2 [file 41419_2026_9144_MOESM1_ESM.docx]

**Sphingosine-1-phosphate ameliorates necrotizing enterocolitis by regulating mitochondrial redox homeostasis and gut microbiota**

**Running title: S1P and NEC**

Wenqiang Sun^1,2,4*^, Yihui Li^1,2,4^, Xue Liu^1,2^, Minqian Zhou^1,2^, Huawei Wang^1^, Haifeng Geng^1^, Wenmei Li^1^, Zhirong Xie^1^, Lili Li^3^, Xinyun Jin^1,2*^, Xueping Zhu^1*^

^1^ Department of Neonatology, Children’s Hospital of Soochow University, Suzhou 215025, China

^2^ Suzhou Medical College, Soochow University, Suzhou 215025, China

^3^ Department of Neonatology, the Affiliated Suzhou Hospital of Nanjing Medical University, Suzhou 215006, China

^4^ These authors contributed equally: Wenqiang Sun and Yihui Li.

**^*^Corresponding Author:**

Wenqiang Sun, [sunwenqiang123456@126.com,](mailto:sunwenqiang123456@126.com,) Department of Neonatology, Children’s Hospital of Soochow University, Suzhou 215025, China.

Xinyun Jin, [jxy150067zzdx@126.com,](mailto:jxy150067zzdx@126.com,) Department of Neonatology, Children’s Hospital of Soochow University, Suzhou 215025, China.

Xueping Zhu, zhuxueping4637@hotmail.com, Department of Neonatology, Children’s Hospital of Soochow University, Suzhou 215025, China.

**Table S1.** Clinical baseline characteristics of the study cohort for metabolomics

| Characteristics | **NEC (n=33)** | **Non-NEC (n=33)** | *P* |
| --- | --- | --- | --- |
| Maternal characteristics |  |  |  |
| Maternal age, years, Median(P25,P75) | 30.00  (27.00, 32.00) | 30.00  (27.50, 33.50) | 0.337 |
| Gestational hypertension (n ,%) | 8 (24.24) | 6 (18.18) | 0.547 |
| Gestational diabetes mellitus (n ,%) | 11 (33.33) | 8 (24.24) | 0.415 |
| PROM>18h (n ,%) | 9 (27.27) | 3 (9.09) | 0.111 |
| Chorioamnionitis (n ,%) | 1 (3.03) | 1 (3.03) | 1.000 |
| Antenatal antibiotics (n ,%) | 12 (36.36) | 8 (24.24) | 0.284 |
| Antenatal MgSO4 (n ,%) | 12 (36.36) | 13 (39.39) | 0.800 |
| Antenatal Steroids (n ,%) | 26 (78.79) | 25 (75.76) | 0.769 |
| Caesarean Section (n ,%) | 15 (45.45) | 13 (39.39) | 0.618 |
| Infant characteristics |  |  |  |
| Female (n ,%) | 19 (57.58) | 17 (51.52) | 0.621 |
| Gestational age (GA), weeks | 29.92±2.06 | 29.71±1.97 | 0.671 |
| Birth weight, g | 1210.91±222.07 | 1174.24±230.22 | 0.513 |
| SGA (n ,%) | 14 (42.42) | 9 (27.27) | 0.196 |
| Postnatal asphyxia (n ,%) | 2 (6.06) | 1 (3.03) | 1.000 |

Necrotizing Enterocolitis (NEC); Prelabor rupture of membranes (PROM); Small for gestational age (SGA)

**Table S2.** Sphingosine-1-phosphate levels in a homogeneous subgroup.

| Characteristics | **NEC (n=15)** | **Non-NEC (n=15)** | *P* |
| --- | --- | --- | --- |
| Maternal age, years, Median(P25,P75) | 31 (28, 35) | 33 (27, 36) | 0.148 |
| Caesarean Section (n ,%) | 6 (40.00) | 7 (46.67) | 0.713 |
| Female (n ,%) | 9 (60.00) | 8 (53.33) | 0.713 |
| Gestational age (GA), weeks | 30.21±0.61 | 30.04±0.70 | 0.811 |
| Birth weight, g | 1346.00±87.82 | 1339.33±61.23 | 0.480 |
| SGA (n ,%) | 5 (33.33) | 5 (33.33) | 1.000 |
| Postnatal asphyxia (n ,%) | 0 | 0 | — |
| S1P (ng/ml) | 65.58±19.89 | 117.20±19.80 | <0.001 |

Necrotizing Enterocolitis (NEC); Small for gestational age (SGA); Sphingosine-1-phosphate (S1P). Neonates in the NEC group were all classified as Bell stage IIB or higher, while the non-NEC group consisted of hospitalized controls without infection.

**Table S3. Primer sequences used for RT-qPCR assays**

| **Species** | **Gene** | **Forward primer (5’-3’)** | **Reverse primer (5’-3’)** |
| --- | --- | --- | --- |
| Mouse/Rat | IL-1β | *AGTGTGGATCCCAAGCAATACCCA* | *TGTCCTGACCACTGTTGTTTCCCA* |
| Mouse/Rat | IL-6 | *GGCTAAGGACCAAGACCATCCAA* | *TCTGACCACAGTGAGGAATGTCCA* |
| Mouse | TNF-α | *GCCTCCCTCTCATCAGTTCTATG* | *ACCTGGGAGTAGACAAGGTACAA* |
| Mouse | β-actin | *TGAGAGGGAAATCGTGCGTGAC* | *GCTCGTTGCCAATAGTGATGACC* |
| Rat | TNF-α | *TTCCGAATTCACTGGAGCCTCGAA* | *TGCACCTCAGGGAAGAATCTGGAA* |
| Rat | S1PR1 | *GCTCTTCATCGTCCTGGTGA* | *AGGATGATGTTGGCAGGTTG* |
| Rat | S1PR2 | *TGGCTGTGGTCTTCATCGTT* | *GCTGATGTTGATGGTGGTGG* |
| Rat | S1PR3 | *CCTGCTACTTCATCGTGGTC* | *AGGATGACGATGAGGATGGA* |
| Rat | β-actin | *TGAAGATCAAGATCATTGCTCCTCC* | *CTAGAAGCATTTGCGGTGGACGATG* |

**Reduced intestinal Sphk2 expression and activity in necrotizing enterocolitis mice**

Compared with controls, sphingosine kinase 1 (SphK1) protein levels in necrotizing enterocolitis (NEC) mice showed no significant change (**Figure S1A**). In contrast, Sphk2 expression was markedly reduced (**Figure S1B**), accompanied by a significant decrease in its enzymatic activity (**Figure S1C**). These findings indicate that both the expression and function of Sphk2 are suppressed under NEC conditions.

**Figure S1.** **Intestinal Sphingosine kinase 2 (Sphk2) protein levels are significantly reduced in necrotizing enterocolitis (NEC) mice. A.** Sphk1 expression in intestinal tissues from the two groups of mice. **B.** Sphk2 expression in intestinal tissues from the two groups of mice. **C.** Relative Sphk2 enzymatic activity in intestinal tissues of the two groups of mice.

**Time-dependent metabolomic remodeling in intestinal tissue following S1P administration**

To systematically characterize the metabolic response following exogenous sphingosine-1-phosphate (S1P) administration, metabolomic profiling was performed on mouse intestinal tissues. Principal component analysis (PCA) revealed clear separation between samples at each time point and the T0 group, indicating that S1P treatment markedly reshaped the global metabolic profile (**Figure S2A**). Pathway enrichment analysis showed that, at the early stage (T1), differential metabolites were predominantly enriched in sphingolipid metabolism and sphingolipid signaling pathways, accompanied by alterations in basal energy metabolism, suggesting rapid activation of lipid signaling networks by exogenous S1P. Notably, sphingolipid metabolism remained significantly enriched across multiple time points, consistent with sustained signaling activation rather than transient substrate turnover (**Figure S2B-F**). With prolonged exposure, enriched pathways gradually shifted toward glycerophospholipid metabolism, unsaturated fatty acid biosynthesis, and fatty acid metabolism, along with changes in oxidative phosphorylation and signal transduction pathways. Time-series clustering analysis further demonstrated distinct temporal dynamics of differential metabolites (**Figure S2G**).

**Figure S2. Time-dependent metabolomic alterations and pathway enrichment in mouse intestinal tissue following sphingosine-1-phosphate (S1P) administration by oral gavage. A.** Principal component analysis (PCA) showing separation of samples across time points (T0-T5). **B-F.** KEGG pathway enrichment analysis of differential metabolites at each time point compared with T0. **G.** Time-series clustering analysis of differential metabolites, grouped into seven subclasses based on dynamic expression patterns across time points. **Notes:** T0, before administration; T1, 30 min post-gavage; T2, 1 h post-gavage; T3, 3 h post-gavage; T4, 6 h post-gavage; T5, 12 h post-gavage.

**Temporal dynamics of sphingolipid-related metabolites and tissue distribution**

At the level of key sphingolipid metabolites, S1P levels increased rapidly at early time points following oral administration, whereas its related metabolites exhibited delayed or fluctuating changes (**Figure S3A-I**), indicating dynamic regulation of the sphingolipid metabolic network across different stages without sustained accumulation. Further quantitative analysis of S1P levels across tissues showed that intestinal S1P rose rapidly after gavage, reached an early peak, and then gradually declined. In contrast, the liver displayed only a mild and delayed increase, while plasma S1P levels remained largely stable throughout the observation period (**Figure S3J-L**). These distribution patterns suggest that exogenous S1P is predominantly enriched locally in the intestine, with minimal entry into systemic circulation.

**Figure S3. Temporal dynamics of sphingolipid-related metabolites and tissue distribution following sphingosine-1-phosphate (S1P) administration by oral gavage. A-I.** Dynamic changes of representative sphingolipid-related metabolites in intestinal tissue at different time points (T0-T5). **J-L.** Quantification of S1P levels in intestinal tissue (J), liver tissue (K), and plasma (L) at corresponding time points. **Notes:** T0, before gavage; T1, 30 min post-gavage; T2, 1 h post-gavage; T3, 3 h post-gavage; T4, 6 h post-gavage; T5, 12 h post-gavage.

**Exogenous S1P accumulates in intestinal epithelial cells and remodels intracellular sphingolipid and glycerophospholipid metabolism**

To directly determine whether exogenous S1P enters intestinal epithelial cells (IECs), IECs were isolated 30 min after S1P gavage, corresponding to the peak intestinal S1P level identified in our previous time-course metabolomic analysis, and further enriched by EpCAM-based magnetic-activated cell sorting (MACS) (**Figure S4A**). ELISA showed that intracellular S1P levels were significantly increased in IECs from the S1P-treated group compared with controls (**Figure S4B**).

Purified IECs were subsequently subjected to untargeted LC–MS/MS metabolomic analysis. OPLS-DA demonstrated a clear separation between the S1P-treated and control groups (**Figure S4C**), indicating that exogenous S1P induced marked intracellular metabolic remodeling following epithelial uptake. A total of 94 differential metabolites were identified, including 33 upregulated and 61 downregulated metabolites (**Figure S4D**). KEGG pathway enrichment analysis revealed that these differential metabolites were predominantly enriched in the sphingolipid metabolism and glycerophospholipid metabolism pathways (**Figure S4E**). Further analysis of key metabolites showed that the sphingolipid-related metabolites SM(d18:1_16:0) and ceramide (d18:1/16:0) were significantly decreased, whereas sphinganine exhibited a decreasing trend that did not reach statistical significance (**Figure S4F–H**). In contrast, intracellular S1P and its degradation product phosphoethanolamine were both significantly increased. In addition, PE(22:6_20:1), a glycerophospholipid metabolite, was also significantly elevated (**Figure S4I–K**).

Collectively, These findings support IECs uptake of exogenous S1P and are accompanied by coordinated remodeling of intracellular sphingolipid and glycerophospholipid metabolism.**Figure S4. Exogenous S1P accumulates in intestinal epithelial cells and remodels intracellular sphingolipid and glycerophospholipid metabolism. A.** Schematic illustration of the experimental workflow. **B.** Intracellular S1P levels in purified intestinal epithelial cells (IECs) measured by ELISA. **C.** OPLS-DA score plot showing metabolic separation between the S1P-treated and control groups. **D.** Volcano plot of differentially abundant metabolites. **E.** KEGG pathway enrichment analysis of differentially abundant metabolites. **F–K.** Relative abundances of representative metabolites, including Sphinganine (F), SM(d18:1_16:0) (G), Ceramide (d18:1/16:0) (H), Sphingosine-1-phosphate (I), Phosphoethanolamine (J), and PE(22:6_20:1) (K).

**S1PR2 and S1PR3 expression levels**

Compared with the control group, neither LPS treatment alone nor co-treatment with S1P or opaganib significantly altered the expression levels of S1PR2 or S1PR3 (**Figure S5A–B**).

**Figure S5. RT-qPCR analysis of S1PR2 (A) and S1PR3 (B) expression levels in IEC-6 cells.**

**Optimization of S1P treatment conditions in vivo and in vitro**

Different doses of S1P administered by gavage exerted distinct effects on survival and intestinal IL-1β levels in NEC mice. A dose of 2 mg/kg significantly improved survival and reduced intestinal IL-1β levels (**Figure S6A-B**). In vitro, S1P improved LPS-induced reductions in IEC-6 cell viability within a certain concentration range, exhibiting both dose- and time-dependent effects. Notably, a significant protective effect was observed at as low as 0.1 μM (**Figure S6C-D**).

**Figure S6. Effects of sphingosine-1-phosphate (S1P) on necrotizing enterocolitis (NEC) mice and LPS-treated IEC-6 cells. A–B.** Effects of different concentrations of S1P gavage intervention on mouse survival rates (**A**) and intestinal IL-1β levels (**B**) after 96 h. **C.** Effects of different S1P (48 h) concentrations on LPS-treated IEC-6 cell viability. **D.** Effects of the S1P (0.1 µM) intervention at different time points on LPS-treated IEC-6 cell viability. **P* <0.05, ***P* < 0.01, ****P* < 0.001

**Optimization of Opaganib treatment conditions in vivo and in vitro**

Different doses of opaganib administered by gavage exerted distinct effects on survival and intestinal IL-1β levels in NEC mice. A dose of 5 mg/kg significantly aggravated mortality and increased intestinal IL-1β levels (**Figure S7A-B**). In vitro, opaganib further reduced LPS-induced IEC-6 cell viability within a certain concentration range, showing both dose- and time-dependent effects. A pronounced effect was observed at 25 μM (**Figure S7C-D**).

**Figure S7. Effects of** **Opaganib on necrotizing enterocolitis (NEC) mice and LPS-treated IEC-6 cells. A–B.** Effects of different concentrations of Opaganib gavage intervention on mouse survival rates (**A**) and intestinal IL-1β levels (**B**) after 96 h. **C.** Effects of different **Opaganib** (48 h) concentrations on LPS-treated IEC-6 cell viability. **D.** Effects of the **Opaganib** (25 µM) intervention at different time points on LPS-treated IEC-6 cell viability. **P* <0.05, ***P* < 0.01, ****P* < 0.001

**Effect of W146 on the viability of IEC-6 cells determined by CCK-8 assay**

CCK-8 assay results showed that treatment with W146 at concentrations of 1, 2.5, 5, and 10 μM did not significantly affect IEC-6 cell viability compared with the control group (*P* > 0.05), indicating that W146 at the tested concentrations does not exert cytotoxic effects and is suitable for functional inhibition experiments (**Figure S8**).

**Figure S8. Effect of W146 on IEC-6 cell viability.** IEC-6 cells were treated with different concentrations of W146 (1, 2.5, 5, and 10 μM), and cell viability was assessed by CCK-8 assay.

**Altered Sphk2 enzymatic activity under different experimental conditions**

Compared with the control group, Sphk2 enzymatic activity was significantly reduced in the intestinal tissues of NEC mice. Under different intervention conditions, Sphk2 activity exhibited corresponding changes (**Figure S9A-D**). These results indicate that Sphk2 activity is dynamically regulated during disease progression and intervention, further supporting its critical role in S1P-related metabolic regulation.

**Figure S9. Measurement of sphk2 enzymatic activity. A.** Sphk2 enzymatic activity in intestinal tissues from two groups of mice. **B.** Sphk2 enzymatic activity in intestinal tissues from four groups of mice. **C.** Sphk2 enzymatic activity in four groups of IEC-6 cells. **D.** Sphk2 enzymatic activity in intestinal tissues from three groups of mice. ***P* < 0.01, ****P* < 0.001
